# Genome analyses reveal the hybrid origin of the staple crop white Guinea yam (*Dioscorea rotundata*)

**DOI:** 10.1073/pnas.2015830117

**Published:** 2020-12-02

**Authors:** Yu Sugihara, Kwabena Darkwa, Hiroki Yaegashi, Satoshi Natsume, Motoki Shimizu, Akira Abe, Akiko Hirabuchi, Kazue Ito, Kaori Oikawa, Muluneh Tamiru-Oli, Atsushi Ohta, Ryo Matsumoto, Paterne Agre, David De Koeyer, Babil Pachakkil, Shinsuke Yamanaka, Satoru Muranaka, Hiroko Takagi, Ben White, Robert Asiedu, Hideki Innan, Asrat Asfaw, Patrick Adebola, Ryohei Terauchi

**Affiliations:** ^a^Laboratory of Crop Evolution, Graduate School of Agriculture, Kyoto University, Kyoto 606-8502, Japan;; ^b^International Institute of Tropical Agriculture, Ibadan 200001, Nigeria;; ^c^Iwate Biotechnology Research Center, Kitakami, Iwate 024-0003, Japan;; ^d^Department of Animal, Plant, and Soil Sciences, School of Life Sciences, La Trobe University, Melbourne, VIC 3086, Australia;; ^e^Fredericton Research and Development Centre, Agriculture and Agri-Food Canada, Fredericton, NB E3B 4Z7, Canada;; ^f^Japan International Research Center for Agricultural Sciences, Tsukuba 305-8686, Japan;; ^g^Department of International Agricultural Development, Tokyo University of Agriculture, Tokyo 183-8538, Japan;; ^h^Earlham Institute, Norwich NR4 7UZ, United Kingdom;; ^i^Laboratory of Population Genetics and Genome Evolution, The Graduate University for Advanced Studies, Hayama 240-0193, Japan

**Keywords:** domestication, Guinea yam, hybrid, population genomics, wild progenitors

## Abstract

White Guinea yam (*Dioscorea rotundata*) is an important staple tuber crop in West Africa. However, its origin remains unclear. In this study, we resequenced 336 accessions of white Guinea yam and compared them with the sequences of wild *Dioscorea* species using an improved reference genome sequence of *D. rotundata*. In contrast to a previous study suggesting that *D. rotundata* originated from a subgroup of *Dioscorea praehensilis*, our results suggest a hybrid origin of white Guinea yam from crosses between the wild rainforest species *D. praehensilis* and the savannah-adapted species *Dioscorea abyssinica*. We identified a greater genomic contribution from *D. abyssinica* in the sex chromosome of Guinea yam and extensive introgression around the *SWEETIE* gene. Our findings point to a complex domestication scenario for Guinea yam and highlight the importance of wild species as gene donors for improving this crop through molecular breeding.

## Significance

Guinea yam is an important staple tuber crop in West Africa, where it contributes to the sustenance and sociocultural lives of millions of people. Understanding the genetic diversity of Guinea yam and its relationships with wild relatives is important for improving this important crop using genomic information. A recent genomics study proposed that Guinea yam originated from a wild relative, the rainforest species *Dioscorea praehensilis*. Our results based on sequencing of 336 Guinea yam accessions do not support this notion; rather, our results indicate a hybrid origin of *Dioscorea rotundata* from crosses between the savannah species *Dioscorea abyssinica* and *D. praehensilis.*

Yams (*Dioscorea* spp.) are major starchy tuber crops that are widely consumed in the tropics. Ten yam species are cultivated worldwide, including *Dioscorea alata* in Southeast Asia, *Dioscorea trifida* in South America, and *Dioscorea rotundata* in West and Central Africa (1). *D. rotundata*, also known as white Guinea yam, is the most important species in West and Central Africa, an area accounting for 92.5% of global yam production in 2018 (http://www.fao.org/statistics). Beyond its nutritional and food value, Guinea yam is also important for the culture of West African people (2).

Despite the considerable importance of Guinea yam, its origin has been elusive. There are two types of Guinea yam: white Guinea yam (*D. rotundata*) and yellow Guinea yam (*Dioscorea cayenensis*). *D. cayenensis* is thought to be a triploid species of hybrid origin, with *D. rotundata* as the maternal parent and *Dioscorea burkilliana* as the paternal parent (3, 4). In turn, the triploid *D. rotundata* is thought to be a hybrid between *D. rotundata* and *Dioscorea togoensis* (4). However, the origin of diploid *D. rotundata*, which accounts for the majority of Guinea yam production (4), has been ambiguous. Two wild species are candidate progenitors of diploid *D. rotundata*: the savannah-adapted wild species *Dioscorea abyssinica* and the rainforest-adapted wild species *Dioscorea praehensilis* (3–10). The geographical distributions of *D. abyssinica* and *D. praehensilis* overlap slightly (*SI Appendix*, Fig. S1). Based on morphological evaluation, Coursey proposed that *D. rotundata* might be a hybrid between the two species (8). However, other reports have indicated that the origin of Guinea yam is ambiguous due to the small number of markers (3–7), introgression (6, 7), or incomplete lineage sorting (7).

The whole-genome sequence of Guinea yam has been reported (11). A recent genome study involving 86 *D. rotundata*, 47 *D. praehensilis*, and 34 *D. abyssinica* accessions suggested that diploid *D. rotundata* was domesticated from *D. praehensilis* (10). Here we addressed this hypothesis using an expanded set of genomes from cultivated and wild *Dioscorea* species.

In this study, we generated an improved version of the Guinea yam reference genome and used it to analyze the genomes of 336 accessions of *D. rotundata* and its wild relatives. Based on these analyses, we attempted to reveal the history of Guinea yam domestication. Our results suggest that diploid *D. rotundata* was most likely derived from homoploid hybridization between *D. abyssinica* and *D. praehensilis*. By evaluating the genomic contributions of each parental species to *D. rotundata*, we revealed greater representation of the *D. abyssinica* genome in the sex chromosome of *D. rotundata* and a signature of extensive introgression in the *SWEETIE* gene on chromosome 17.

## Genetic Diversity of Guinea Yam

**Fig. 1.**
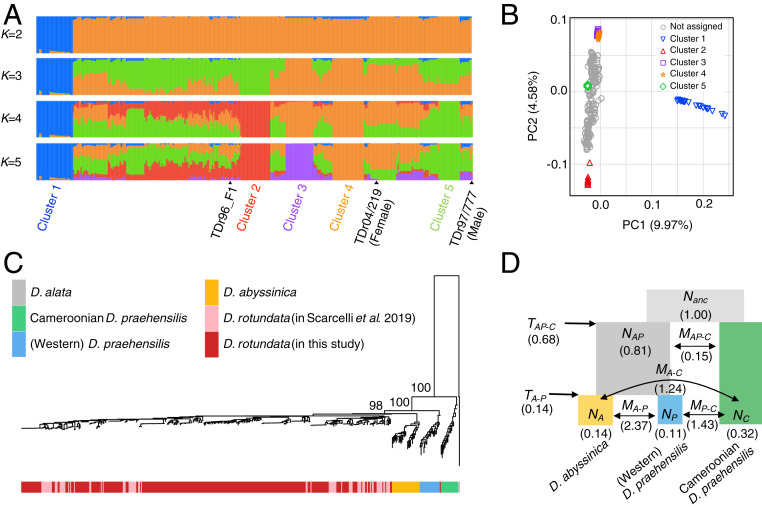
Genetic diversity and phylogenomics of Guinea yam and its wild relatives. (*A*) Ancestry proportions of each Guinea yam accession with 6,124,093 SNPs. TDr96_F1 is the sample used as the reference genome. (*B*) PCA result of the 336 Guinea yam accessions. (*C*) NJ tree of four African yam lineages reconstructed using *D. alata* as an outgroup based on 463,293 SNPs. The numbers indicate bootstrap values after 100 replications. The sequences of *D. rotundata* in the previous study (10) were included in the tree. (*D*) Evolutionary relationship of three African wild yam lineages (*D. abyssinica*, Western *D. praehensilis*, and Cameroonian *D. praehensilis*) as inferred by ∂a∂i (15) using 17,532 SNPs. *N*, *M*, and *T* represent the relative population size from *N*_*anc*_, migration rate, and divergence time, respectively.

We obtained DNA samples from 336 accessions of *D. rotundata* maintained at the International Institute of Tropical Agriculture (IITA) in Nigeria, representing the genetic diversity of Guinea yam landraces and improved lines from West Africa. We subjected these samples to whole-genome resequencing on the Illumina sequencing platform. We aligned the resulting short reads to the newly assembled reference genome (*SI Appendix*, sections S1 and S2) and extracted single nucleotide polymorphism (SNP) information for use in genetic diversity studies (*SI Appendix*, Table S1 and section S3). Based on admixture analysis with the sNMF program (12), we defined five major clusters (Fig. 1*A*). When *K* = 2, cluster 1 was clearly separated from the other accessions. Principal component analysis (PCA) also separated cluster 1 from the rest of the clusters (Fig. 1*B*). Accessions in cluster 1 had significantly higher heterozygosity and ∼10-fold more unique alleles than those in the four remaining clusters (*SI Appendix*, Figs. S2 and S3 and Table S2). Because flow cytometry analysis confirmed that all 10 accessions analyzed in cluster 1 were triploids (Dataset S1), we hypothesized that cluster 1 represents triploid *D. rotundata*, a hybrid of *D. rotundata* and *D. togoensis* (4). After removing the cluster 1 accessions, the nucleotide diversity of *D. rotundata* was estimated as 14.83 × 10^−4^ (*SI Appendix*, Table S3), which is ∼1.5-fold larger than that reported previously (10), presumably because we used a larger number of samples with diverse genetic backgrounds in our study. Linkage disequilibrium of diploid *D. rotundata* showed a decay of *r*^2^ = 0.13 in a 200-kb genomic region (*SI Appendix*, Fig. S4), which is slower than that of cassava, another clonally propagated crop (13).

## Phylogenomic Analysis of African Yam

Using the SNP information, we constructed a rooted neighbor-joining (NJ) tree (14) based on 308 Guinea yam accessions sequenced in the present study (excluding cluster 1 triploid accessions), as well as 80 *D. rotundata*, 29 *D. abyssinica*, 21 Western *D. praehensilis*, and 18 Cameroonian *D. praehensilis* accessions that were sequenced in a previous study (10) using two accessions of Asian species *D. alata* as an outgroup (Fig. 1*C*). Throughout the analyses described below, we used 388 *D. rotundata* accessions by combining our samples and those used previously (10). According to this NJ tree, the *D. rotundata* accessions sequenced in this study are genetically close to the *D. rotundata* accessions reported previously (10) (Fig. 1*C*). However, the NJ tree showed that *D. rotundata* is more closely related to *D. abyssinica* than to Western *D. praehensilis* (Fig. 1*C*), which is inconsistent with a previous finding (10) that *D. rotundata* is most closely related to Western *D. praehensilis*.

To elucidate the evolutionary relationships of the three wild *Dioscorea* species that are closely related to *D. rotundata*—*D. abyssinica* (designated as A), Western *D. praehensilis* (P), and Cameroonian *D. praehensilis* (C)—we performed diffusion approximations for demographic inference (∂a∂i) analysis (15), which allows for estimation of demographic parameters based on an unfolded site frequency spectrum. First, we tested three phylogenetic models—{{A, P}, C}, {{P, C}, A}, and {{C, A}, P}—using 17,532 SNPs that were polarized using *D. alata* as an outgroup without considering migration among the species. Of the three models, {{A, P}, C} had the highest likelihood (*SI Appendix*, Table S4).

This result is not consistent with the previous finding that {{P, C}, A} had the highest likelihood (10), as determined using a different method with fastsimcoal2 software (16). To exactly repeat the previous analysis, we tested these three models with fastsimcoal2 (16) using the previous reference genome (11), which indicated that {{A, P}, C} had the highest likelihood (*SI Appendix*, Table S5). Taken together, our results are inconsistent with the previous report (10) but are consistent with the PCA result from the same report, which separated Cameroonian *D. praehensilis* from the other African yams in PC1 (figure 2A of ref. 10).

Based on the assumption that {{A, P}, C} describes the true evolutionary relationship among the three wild *Dioscorea* species, we reestimated the evolutionary parameters with ∂a∂i, allowing symmetric migration (gene flow) among the species (Fig. 1*D*). Since the results indicated that Cameroonian *D. praehensilis* is distantly related to *D. rotundata* and was not likely involved in genetic exchange with *D. rotundata* (Fig. 1*C*), we focused on Western *D. praehensilis*, which we refer to as *D. praehensilis* hereinafter for brevity.

## Hybrid Origin of Guinea Yam

**Fig. 2.**
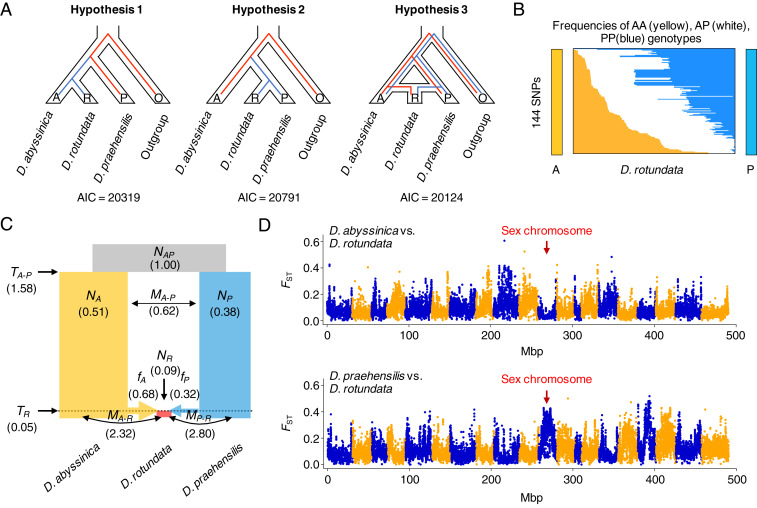
Evidence for the hybrid origin of Guinea yam. (*A*) Hypotheses for the domestication of Guinea yam (*D. rotundata*). Hypothesis 1 assumes that *D. rotundata* diverged from *D. abyssinica*. Hypothesis 2 assumes that *D. rotundata* diverged from *D. praehensilis*. Hypothesis 3 assumes that *D. rotundata* was derived from a hybrid between *D. abyssinica* and *D*. *praehensilis*. *D. alata* served as an outgroup. (*B*) Frequencies of individuals homozygous for *D. abyssinica* allele (A, in yellow), homozygous for *D. praehensilis* allele (P, in blue), and heterozygous for A and P (in white) among the 388 *D. rotundata* sequences as studied for 144 SNPs. (*C*) Evolutionary parameters related to the hybrid origin of Guinea yam as inferred by ∂a∂i (15) using 15,461 SNPs. *N*, *M*, and *T* represent the relative population size from *N*_*AP*_, migration rate, and divergence time, respectively. *f*_*A*_ and *f*_*p*_ indicate the genomic contributions from *D. abyssinica* and *D. praehensilis* when the hybridization occurred, respectively. (*D*) *F*_*ST*_ between the wild and cultivated yams. This was conducted with a 100-kb window and a 20-kb step. Chromosome 11 of *D. rotundata* containing the sex-determining locus shows a shorter distance to that of *D. abyssinica* and a longer distance to that of *D. praehensilis*.

We propose three hypotheses for the origin of Guinea yam (*D. rotundata*) based on the NJ tree (Fig. 1*C*) and ∂a∂i (15) (Fig. 1*D*). The first hypothesis is that *D. rotundata* was derived from *D. abyssinica* (hypothesis 1 in Fig. 2*A*); the second is that *D. rotundata* was derived from *D. praehensilis* (hypothesis 2 in Fig. 2*A*). However, in hypotheses 1 and 2, the divergence time of *D. rotundata* from the wild species might not be sufficient to separate the three lineages, and there may be incomplete lineage sorting among the species. The third hypothesis is that *D. rotundata* originated as an admixture between *D. abyssinica* and *D. praehensilis* (hypothesis 3 in Fig. 2*A*).

Before estimating the evolutionary parameters for the three hypotheses, we studied the allele frequencies of the 388 *D. rotundata* sequences, focusing on 144 SNPs that are positioned over the entire genome and are oppositely fixed in the two candidate progenitors (Fig. 2*B* and *SI Appendix*, Fig. S5). If hypothesis 1 or 2 is correct, then the allele frequencies in these 144 SNPs should be highly skewed to either of the progenitors. The patterns of allele contributions from the two candidate species to *D. rotundata* were nearly identical, however. This result suggests that hypothesis 3, the admixture origin of Guinea yam, is most likely correct.

We tested the three hypotheses by ∂a∂i (15) with symmetric migration (gene flow) rates using 15,461 SNPs polarized by *D. alata* (Fig. 2*A*), which showed that hypothesis 3 had the highest likelihood and the lowest Akaike information criterion value (Fig. 2*C* and *SI Appendix*, Table S4). This result supports the admixture hypothesis, that *D. rotundata* was derived from crosses between *D. abyssinica* and *D. praehensilis*. The parameters estimated by ∂a∂i indicate that the hybridization between *D. abyssinica* and *D. praehensilis* was relatively recent in relation to the divergence between the two wild species. This analysis also indicated that the genomic contributions from *D. abyssinica* and *D. praehensilis* during the hybridization period were ∼68% and 32%, respectively. Introgression generally results in highly asymmetric genomic contributions from the parental species, whereas hybridization shows symmetric genomic contributions (17). The intermediate genomic contributions revealed by this analysis support the hybridization hypothesis rather than the introgression hypothesis. Our findings are in line with the hybrid origin of the Guinea yam proposed by Coursey in 1976 based on morphology (8) and supports his speculation that spontaneous hybridization between wild yams could have occurred at the artifactual “dump heaps” created by people living in the savannah between the forest and the Sahara (9).

To evaluate the genetic distances of *D. rotundata* from the two parental species for each chromosome, we calculated fixation index (*F*_*ST*_) values (18) (Fig. 2*D* and *SI Appendix*, Table S6). The genetic distances from the two parents varied across the different chromosomes, and the overall genetic distance of *D. rotundata* from *D. abyssinica* was shorter than that from *D. praehensilis* (*SI Appendix*, Table S6). Intriguingly, chromosome 11, to which we previously mapped the candidate locus for sex determination (11), had the shortest genetic distance from *D. abyssinica* and the longest genetic distance from *D. praehensilis* among all chromosomes, indicating that chromosome 11 of *D. rotundata* is highly skewed to *D. abyssinica* (Fig. 2*D* and *SI Appendix*, Table S6). Similarly, interspecies divergence is different between the autosomes and sex chromosome of the dioecious plant species *Silene* (19).

## Evolutionary History of Guinea Yam

**Fig. 3.**
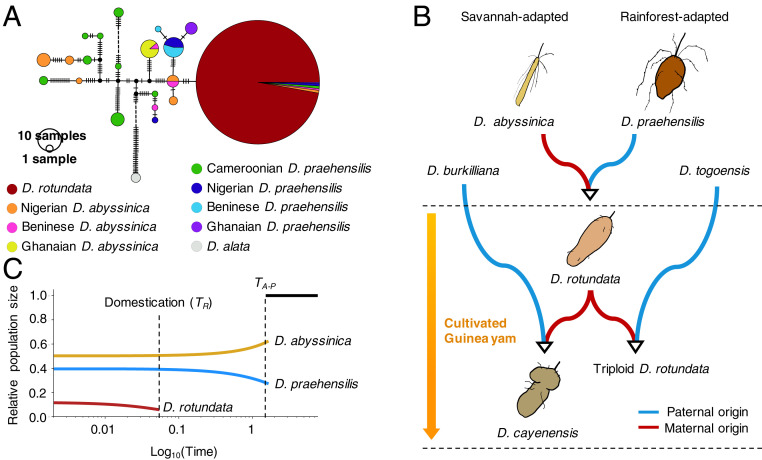
Evolutionary scenario of African yam origins. (*A*) Haplotype network of the whole plastid genomes of 416 *D. rotundata* (including the triploid accessions), 68 wild relatives, and two *D. alata* accessions as the outgroup. The number of vertical dashes represents the number of mutations. Western (Nigerian, Beninese, and Ghanaian) *D. praehensilis* and *D. rotundata* seem to have diverged from Nigerian and Beninese *D. abyssinica*. (*B*) Possible scenario of domestication of Guinea yam. The blue line represents paternal origin, and the red line represents maternal origin. (*C*) Changes in population sizes of *D. rotundata* and its wild relatives as inferred by ∂a∂i (15). The parameters except population size were identical to those used in Fig. 2*C*. After the domestication of *D. rotundata*, the population size of *D. rotundata* has increased with migration from the wild progenitors.

In angiosperms, plastid genomes are predominantly inherited maternally (20), making them useful for studying maternal lineages. To infer the maternal history of Guinea yam, we constructed a haplotype network of the whole plastid genome with all samples used in the NJ tree (Fig. 1*C*), as well as the triploid accessions in cluster 1 (Fig. 3*A* and *SI Appendix*, section S6). According to this haplotype network, Cameroonian *D. praehensilis* has the longest genetic distance from *D. rotundata*. This result is in line with the phylogenomic trees of African yam (Fig. 1 *C* and *D*). Strikingly, the plastid genomes of diploid and triploid *D. rotundata* are uniform and very similar to those of Nigerian or Beninese *D. abyssinica,* although the latter has another plastid genome lineage distant from that of *D. rotundata*. The plastid genomes of *D. praehensilis* from Nigeria, Benin, and Ghana appear to be derived from Nigerian or Beninese *D. abyssinica*. These results indicate that *D. abyssinica* is an older lineage than *D. praehensilis*, and that the places of origin of *D. rotundata* and *D. praehensilis* are probably around Nigeria or Benin. Based on the whole-genome diversity of *D. rotundata*, a recent study (10) hypothesized that the origin of *D. rotundata* was around north Benin, as supported by the current results. The plastid genomes of some wild species are identical to those of cultivated Guinea yams. Hybridization between cultivated yams and wild yams may account for this observation (7).

The results of nuclear genome admixture (Fig. 2) and plastid haplotype network (Fig. 3*A*) analyses indicate that the maternal origin of diploid *D. rotundata* is *D. abyssinica* and its paternal origin is *D. praehensilis* (Fig. 3*B*). Hybridization between *D. abyssinica* and *D. praehensilis* is rare (10), but such rare hybrids appear to have been domesticated by humans. The triploid *D. rotundata* shares its plastid haplotype with diploid *D. rotundata*, indicating that diploid *D. rotundata* served as the maternal parent and *D. togoensis* was the paternal parent. *D. cayenensis* is reported to have *D. rotundata* as the maternal parent and *D. burkilliana* as the paternal parent (3, 4). All cultivated Guinea yams are hybrids containing *D. abyssinica* plastid genomes.

To explore the changes in population size, we reinferred the demographic history of African yam by ∂a∂i (15), allowing migration (Fig. 3*C* and *SI Appendix*, section S7). We used the same dataset as in Fig. 2*C*. By fixing the parameters predicted in Fig. 2*C* except population size, we reestimated each population size at the start and end points after the emergence of these species, assuming an exponential increase/decrease in population size. According to this analysis, since the emergence of the wild progenitors of Guinea yam, the population size of *D. abyssinica* has been decreasing, while that of *D. praehensilis* has been increasing (Fig. 3*C*). This finding suggests that the *D. praehensilis* population was derived from *D. abyssinica*, which is consistent with the results of haplotype network analysis (Fig. 3*A*).

## Extensive Introgression at the *SWEETIE* Locus

**Fig. 4.**
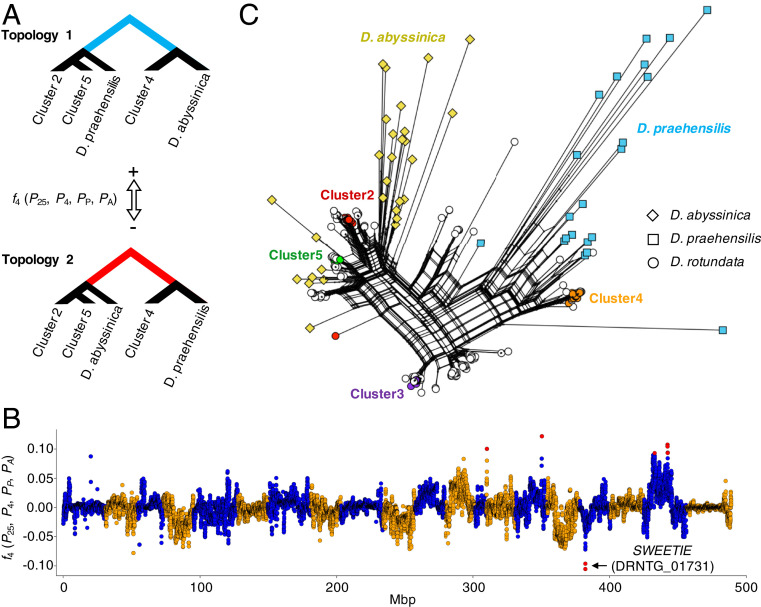
Signature of extensive introgression around the *SWEETIE* gene. (*A*) Topology of *f*_*4*_ (*P*_*25*_*, P*_*4*_*, P*_*P*_*, P*_*A*_) in clusters 2, 4, and 5 and wild yams. Positive *f*_*4*_ values represent the long internal branch of the upper tree (topology 1), and negative *f*_*4*_ values represent the long internal branch of the bottom tree (topology 2). (*B*) *f*_*4*_ values across the genome. This was conducted with a 250-kb window and a 25-kb step. Red dots indicate outliers of the sliding window, which have |*Z*(*f*_*4*_)| > 5. The locus around the *SWEETIE* gene shows extraordinarily negative *f*_*4*_ values. (*C*) Neighbor-Net around the *SWEETIE* gene (4∼4.15 Mb on chromosome 17). This was constructed by SplitsTree (22) using a total of 458 SNPs.

To explore multiple introgression to *D. rotundata* from the two wild species, we analyzed the *f*_4_ statistic (21) using four groups: *D. rotundata* clusters 2 and 5, *D. rotundata* cluster 4, *D. abyssinica*, and *D. praehensilis* (*SI Appendix*, section S8). The *f*_4_ statistic reveals the representation of two alternative discordant genealogies (Fig. 4*A*). The *f*_4_ value is close to zero if the first two groups of *D. rotundata* show a concordant genealogy in relation to *D. abyssinica* and *D. praehensilis*. In contrast, the *f*_4_ value diverges from zero if the two groups of *D. rotundata* exhibit discordant genealogy and a large genetic distance to each other. We obtained the *f*_4_ statistic *f*_4_ (*P*_25_, *P*_4_, *P*_P_, *P*_A_) for each SNP and performed sliding window analysis (Fig. 4*B*). The *f*_4_ value was close to zero across the genome, indicating that overall, we cannot decide between topology 1 and topology 2. However, the genomic regions around the *SWEETIE* gene showed the lowest *f*_4_ (*P*_25_, *P*_4_, *P*_P_, *P*_A_) [*Z*(*f*_4_) = −5.66], with overrepresentation of topology 2 in the *SWEETIE* gene (DRNTG_01731) (*SI Appendix*, Table S7).

To explore the genealogical relationships around the *SWEETIE* gene, we constructed a Neighbor-Net (22) around this locus (4.00 to 4.15 Mb on chromosome 17) (Fig. 4*C*). The Neighbor-Net showed that the locus of cluster 4 was close to that of *D. praehensilis*, while the loci of clusters 2 and 5 and some other accessions were close to those of *D. abyssinica*. These results indicate that the *SWEETIE* gene was introgressed from the wild species more than once. The *SWEETIE* gene encodes a membrane protein involved in the general control of sugar flux (23). The *Arabidopsis thaliana sweetie* mutant shows pronounced changes in the accumulation of sugar, starch, and ethylene along with significant changes in growth and development (24). We still do not know the effect of this introgression on the phenotype of Guinea yam, but this locus appears to be a target of selection.

## Homoploid Hybrid Formation as the Trigger of Domestication

The importance of hybridization and polyploidization for crop domestication is well documented (25, 26), including in bread wheat (27) and banana (28). Compared with allopolyploidy, only a limited number of homoploid hybridizations have been reported in plants (29), and homoploid hybridizations have rarely contributed to the origin of crops (30). Homoploid hybridization can increase genetic variation via recombination between distantly related species, and it often allows the hybrid to adapt to unexploited niches (31). In the case of Guinea yam, the savannah-adapted wild species *D. abyssinica* and the rainforest-adapted wild species *D. praehensilis* are not suitable for agriculture; however, their hybrid, *D. rotundata*, could have been adopted for cultivation by humans. Gene combinations from different wild yams might have contributed to the domestication of Guinea yam. The present study provides an example of the origin of a crop through homoploid hybridization.

## Use of Wild Species to Improve Guinea Yam

A project for the improvement of Guinea yam by crossbreeding has been initiated (AfricaYam: https://africayam.org). However, the current breeding projects depend solely on *D. rotundata* genetic resources. Systematic efforts are needed to introgress beneficial alleles from wild species into crops; these alleles will increase disease resistance and abiotic stress tolerance to improve crop resiliency and productivity (32). Our study revealed that the two wild progenitor species (*D. abyssinica* and *D. praehensilis*) of Guinea yam contain much greater genetic diversity than *D. rotundata* (Fig. 2*C*), suggesting that these wild species could be useful sources for alleles of agricultural importance. However, the *D. abyssinica* and *D. praehensilis* accessions in IITA GenBank account for only 1.6% of the total *Dioscorea* accessions maintained as of 2018 (33). Therefore, it will be important to collect and preserve wild *Dioscorea* species as genetic resources for improving Guinea yam. Our findings suggest that new alleles of loci, such as the *SWEETIE* gene, were introgressed from wild yams into cultivated Guinea yams multiple times, which likely conferred the plants with phenotypes preferred by humans. Many more alleles from wild species remain to be exploited for systematic breeding. Our findings highlight the need to consider how to effectively leverage the gene pools of wild species from different habitats for the rapid breeding of Guinea yam using genomic information.

## Supplementary Material

Supplementary File

Supplementary File

## Data Availability

All study data are included in the main text and supporting information.
