# Supplementary material for: Genome analyses reveal the hybrid origin of the staple crop white Guinea yam (Dioscorea rotundata)
Source: Proc Natl Acad Sci U S A. 2020 Dec 2;117(50):31987–92. doi: 10.1073/pnas.2015830117 (PMC7749330; doi:10.1073/pnas.2015830117)
Supplement: Supplementary File [file pnas.2015830117.sapp.pdf]

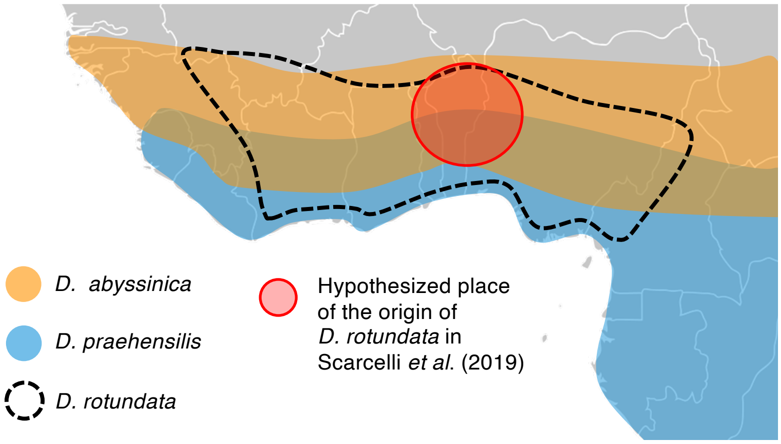

Fig. S1. The geographical distributions of African yams. Adapted from Scarcelli *et al*. (2017) (*40*) and Scarcelli *et al*. (2019) (*28*).

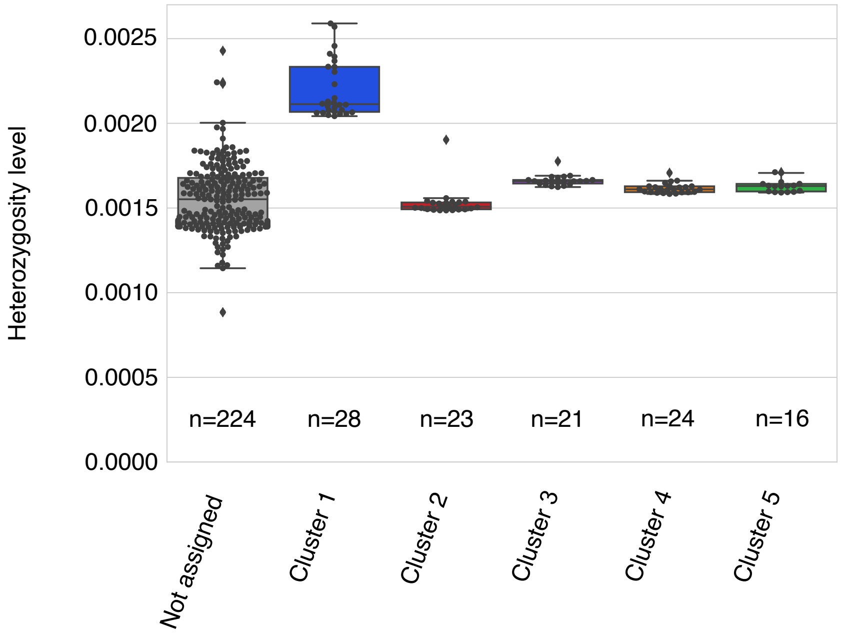

Fig. S2. Heterozygosity levels of samples in five clusters of *D. rotundata*. Heterozygosity level of an individual is defined as the ratio of number of heterozygous SNPs to the total number of mapped sites to the reference genome.

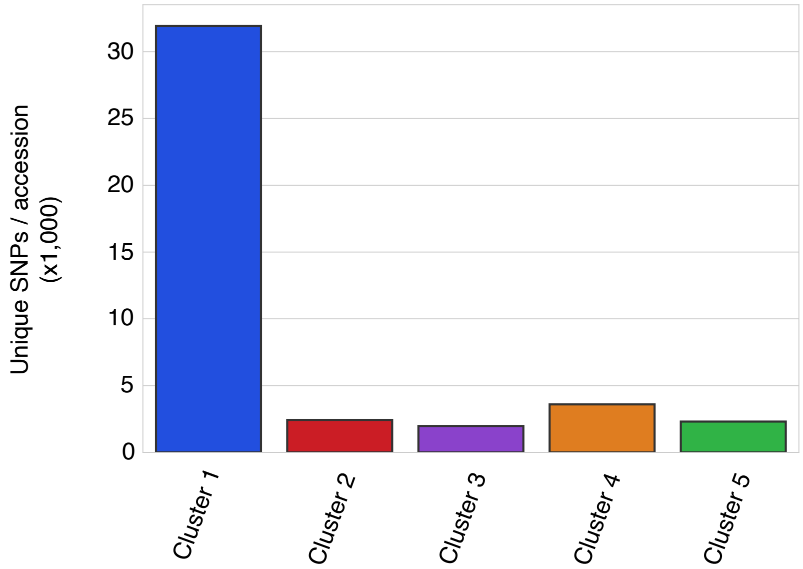

Fig. S3. Number of unique alleles in the five clusters of *D. rotundata*.

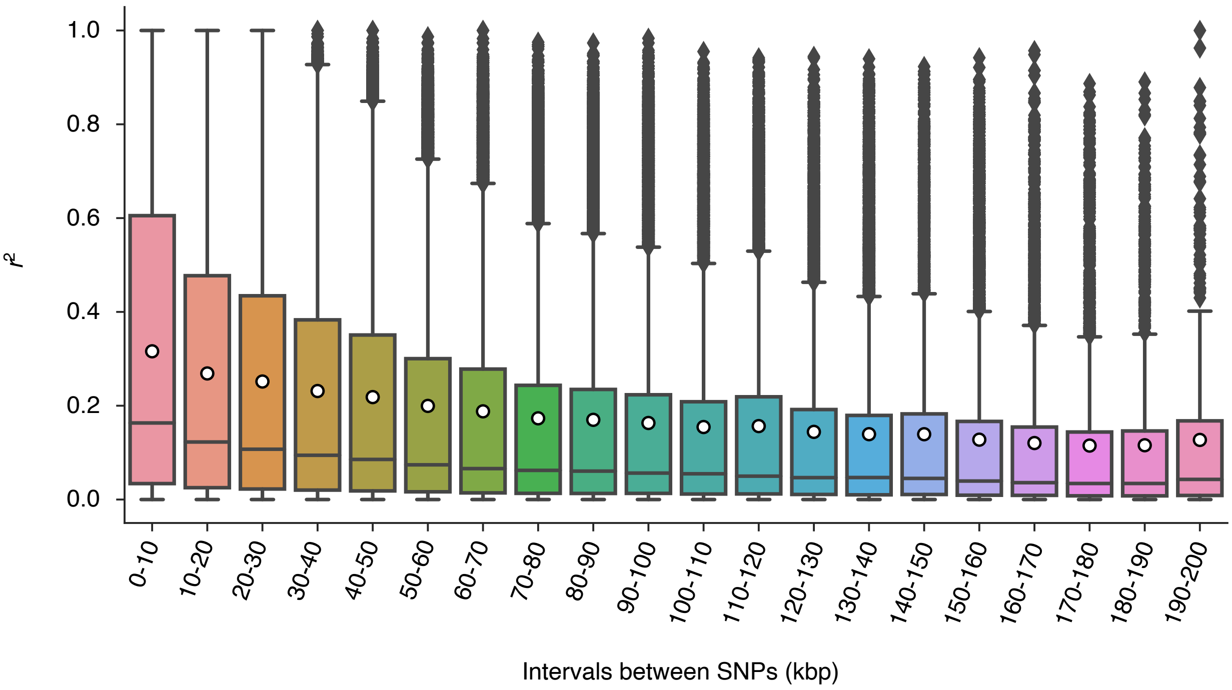

Fig. S4. LD decay of *D. rotundata*. Each white dot represents the average r^2 in each interval.

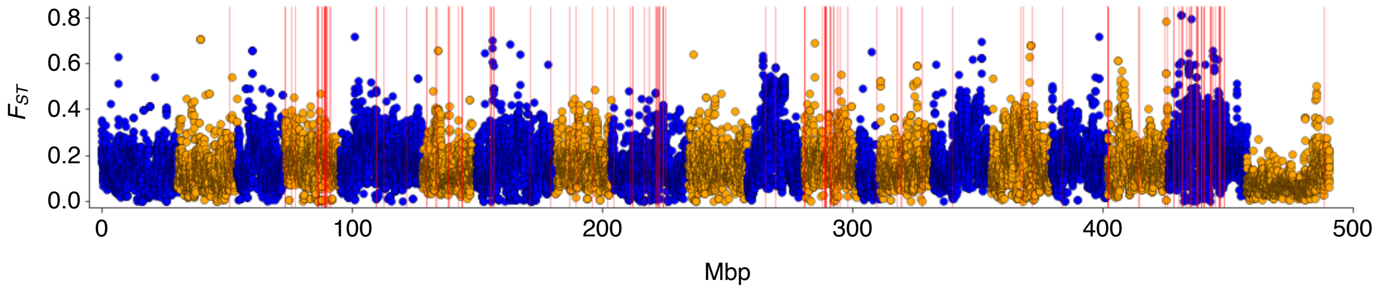

Fig. S5. **F_{ST} between *D. abyssinica* and *D. praehensilis*.** F_{ST} averages were calculated 100-kb window and 20-kb step. The red vertical lines represent the positions of the oppositely fixed SNPs in *D. abyssinica* and *D. praehensilis* as used in Fig. 2B.

Table S1. For each Figure and Table, the number of SNPs studied, use of triploid *D. rotundata* samples (cluster 1), and use of Scarcelli's samples are indicated.

Figures/tables	No. analyzed SNPs	Triploid *D. rotundata* (in cluster 1)	Dioploid *D. rotundata* (not in cluster 1)	Samples in Scarcelli *et al.* 2019
Fig. 1A and B, Fig. S2 and S3, Table S2	6,124,093	yes	yes	-
Table S3	5,229,368	no	yes	-
Fig. 1C	463,293	no	yes	C/A/P/R
Fig. 1D	17,532	no	no	C/A/P
Table S5	87,671	no	no	C/A/P
Fig. 2B	144	no	yes	A/P/R
Fig. 2C and 3C	15,461	no	yes	A/P/R
Fig. 2D (A vs. R)	649,679	no	yes	A/R
Fig. 2D (P vs. R)	579,405	no	yes	P/R
Fig. S5 (A vs. P)	362,125	no	no	A/P
Fig. 3A	250	yes	yes	C/A/P/R
Fig. 4B	2,343,307	no	yes	A/P
Fig. 4C	458	no	yes	A/P

C: Cameroonian *D. praehensilis*
A: *D. abyssinica*
P: (Western) *D. praehensilis*
R: *D. rotundata*

Table S2. Comparison of heterozygosity levels in the five clusters of *D. rotundata*. Heterozygosity level of an individual is defined as the ratio of number of heterozygous SNPs to the total number of mapped sites to the reference genome. The diagonal cell represents the mean ± standard deviation of the heterozygosity levels of the samples in each cluster. The other cells represent *P*-values of the difference of the heterozygosity levels between the two clusters as calculated by two-tailed Student t test. Cluster 1 has a significantly higher heterozygosity level than the other clusters.

	**Not assigned**					
**Not assigned**	15.53×10^{-4} ($\pm1.96\times10^{-4}$)	**Cluster 1**				
**Cluster 1 (n=28)**	2.874×10^{-42}	21.98×10^{-4} ($\pm1.68\times10^{-4}$)	**Cluster 2**			
**Cluster 2 (n=23)**	0.5483	1.453×10^{-22}	15.29×10^{-4} ($\pm0.84\times10^{-4}$)	**Cluster 3**		
**Cluster 3 (n=21)**	0.01194	8.305×10^{-19}	2.582×10^{-8}	16.62×10^{-4} ($\pm0.32\times10^{-4}$)	**Cluster 4**	
**Cluster 4 (n=24)**	0.1188	4.358×10^{-22}	1.759×10^{-5}	9.915×10^{-6}	16.16×10^{-4} ($\pm0.30\times10^{-4}$)	**Cluster 5**
**Cluster 5 (n=16)**	0.1203	1.344×10^{-16}	6.272×10^{-5}	7.857×10^{-3}	0.1972	16.30×10^{-4} ($\pm0.37\times10^{-4}$)

Table S3. Population genetics summary statistic in the 308 yam accessions

	After imputation
No. segregating site	5,229,368
No. singleton	1,227,900
θ_W	14.98 x 10^{-4}
θ_π	14.83 x 10^{-4}
Tajima's *D*	-0.0305

Table S4. Likelihood comparison in ∂a∂i

Model	$\log_{10}(L)$	No. parameters	AIC	Illustration of the model
{{A, P}, C} (without migration)	-15289.70	6	30591.40	-
{{P, C}, A} (without migration)	-15765.32	6	31542.64	-
{{C, A}, P} (without migration)	-15765.15	6	31542.29	-
{{A, P}, C} (with migration)	-12739.86	10	25499.72	Fig. 1D
{{A, R}, P} (with migration)	-10149.73	10	20319.47	-
{{P, R}, A} (with migration)	-10385.46	10	20790.92	-
{{A, R}, {P, R}} (with migration)	-10052.96	9	20123.91	Fig. 2C
{{A, R}, {P, R}}<br>- With migration<br>- With population growth<br>- Fix the parameters except for population size	-10046.73	6	20105.47	Fig. 3C

C: Cameroonian *D. praehensilis*
A: *D. abyssinica*
P: (Western) *D. praehensilis*
R: *D. rotundata*

Table S5. Likelihood comparison in fastsimcoal2

Model	$log_{10}(L)$
{{A, P}, C} (without migration)	-172110.065
{{P, C}, A} (without migration)	-174281.072
{{C, A}, P} (without migration)	-173358.592

Table S6. F_{ST} in each chromosome. Red and blue indicates the highest and lowest F_{ST} in all chromosomes, respectively. Chromosome 11 of *D. rotundata* containing the sex-determining locus shows a lower distance to that of *D. abyssinica*.

	A vs. P		A vs. R		P vs. R	
**Chromosome**	F_{ST}	**± std**	F_{ST}	**± std**	F_{ST}	**± std**
**All**	0.162	0.217	0.082	0.120	0.123	0.157
**chrom_01**	0.156	0.222	0.079	0.109	0.084	0.112
**chrom_02**	0.122	0.187	0.055	0.078	0.098	0.121
**chrom_03**	0.177	0.224	0.075	0.103	0.101	0.115
**chrom_04**	0.173	0.218	0.111	0.150	0.100	0.130
**chrom_05**	0.201	0.257	0.098	0.128	0.115	0.133
**chrom_06**	0.116	0.168	0.065	0.092	0.075	0.102
**chrom_07**	0.161	0.231	0.093	0.122	0.084	0.114
**chrom_08**	0.165	0.209	0.120	0.161	0.085	0.109
**chrom_09**	0.129	0.170	0.129	0.150	**0.062**	0.102
**chrom_10**	0.152	0.205	0.129	0.169	0.077	0.102
**chrom_11**	**0.277**	0.273	**0.033**	0.052	**0.247**	0.231
**chrom_12**	0.160	0.213	0.063	0.096	0.134	0.140
**chrom_13**	0.111	0.161	0.064	0.100	0.108	0.120
**chrom_14**	0.141	0.184	0.120	0.163	0.107	0.133
**chrom_15**	0.204	0.243	**0.133**	0.152	0.073	0.104
**chrom_16**	0.192	0.248	0.050	0.074	0.174	0.182
**chrom_17**	0.180	0.201	0.062	0.080	0.217	0.221
**chrom_18**	0.169	0.210	0.074	0.103	0.188	0.205
**chrom_19**	0.191	0.240	0.080	0.110	0.133	0.152
**chrom_20**	**0.070**	0.109	0.057	0.088	0.126	0.143

Table S7. List of genes in the five outlier loci (chromosome 14, 15, 17, 19) showing extreme f_4 (P_{25}, P_4, P_P, P_A) values (|Z(f_4)| > 5) in Figure 4B.

Chromosome	Start	End	GeneID	Annotation
chrom_14	468088	469472	DRNTG_17186.1	(TrEMBL)Predicted protein(HORVV:F2DKZ3)
chrom_14	484029	484961	DRNTG_28166.1	(TrEMBL)Uncharacterized protein(ENSVE:A0A444CGI1)
chrom_14	485725	490867	DRNTG_28165.1	(TrEMBL)Endoplasmic reticulum metallopeptidase 1(ANACO:A0A199W086)
chrom_14	492377	496008	DRNTG_28164.1	Auxin response factor 18(ORYSJ:Q653H7)
chrom_14	496093	496525	DRNTG_28163.1	-
chrom_14	501391	506132	DRNTG_28162.1	Protein ENHANCED DISEASE RESISTANCE 2(ARATH:F4JSE7)
chrom_14	507961	513788	DRNTG_28161.1	Clathrin interactor EPSIN 2(ARATH:Q67YI9)
chrom_14	514348	516233	DRNTG_28160.1	Mitochondrial import inner membrane translocase subunit PAM16 like 2(ARATH:Q93VV9)
chrom_14	516747	519058	DRNTG_28159.1	Cytokinin riboside 5'-monophosphate phosphoribohydrolase LOG4(ARATH:Q9LFH3)
chrom_14	520890	523855	DRNTG_28157.1	Protein CNGC15c(MEDTR:A0A072VMJ3)
chrom_14	521076	521734	DRNTG_28158.1	Protein CNGC15c(MEDTR:A0A072VMJ3)
chrom_14	527056	529173	DRNTG_28156.1	Phytochrome-associated serine/threonine-protein phosphatase(PEA:Q8LSN3)
chrom_14	531504	532632	DRNTG_11714.1	-
chrom_14	544864	552211	DRNTG_11716.1	Phenylalanine ammonia-lyase 3(PETCR:P45729)
chrom_14	550860	554840	DRNTG_11717.1	Phenylalanine ammonia-lyase 3(PETCR:P45729)
chrom_14	565849	567237	DRNTG_11718.1	(TrEMBL)Uncharacterized protein(SETIT:K3ZZF5)
chrom_14	581692	585798	DRNTG_11720.1	E3 ubiquitin-protein ligase WAV3(ARATH:Q9LTA6)
chrom_14	586418	589346	DRNTG_11721.1	General transcription factor IIH subunit 2(ARATH:Q9ZVN9)
chrom_14	589956	591825	DRNTG_11722.1	Probable mannitol dehydrogenase(FRAAN:Q9ZRF1)
chrom_14	607695	608572	DRNTG_25842.1	-
chrom_14	612414	613708	DRNTG_25841.1	(TrEMBL)Uncharacterized protein(MUSAM:M0SPY3)
chrom_14	624755	628872	DRNTG_25840.1	Phenylalanine ammonia-lyase 3(PETCR:P45729)
chrom_14	632310	633887	DRNTG_25839.1	Phenylalanine ammonia-lyase(BROFI:Q42609)
chrom_14	648000	649003	DRNTG_25837.1	-
chrom_14	681229	685056	DRNTG_20830.1	-
chrom_14	695533	696149	DRNTG_12014.1	Ferredoxin--NADP reductase, embryo isozyme, chloroplastic(ORYSJ:O23877)
chrom_14	696349	702726	DRNTG_12015.1	Serine--tRNA ligase, cytoplasmic(ARATH:Q39230)
chrom_14	538878	542983	DRNTG_11715.1	Adenosine kinase 2(ARATH:Q9LZG0)
chrom_14	571194	575569	DRNTG_11719.1	Synaptotagmin-3(ARATH:Q7XA06)
chrom_14	604820	608527	DRNTG_25843.1	Synaptotagmin-3(ARATH:Q7XA06)
chrom_14	637226	641558	DRNTG_25838.1	Adenosine kinase 2(ARATH:Q9LZG0)
chrom_14	717990	726467	DRNTG_12016.1	Adenosine kinase 1(ARATH:Q9SF85)
chrom_15	19356271	19357116	DRNTG_00821.1	-
chrom_15	19362481	19363591	DRNTG_00822.1	-
chrom_15	19603544	19604222	DRNTG_00824.1	ADP,ATP carrier protein, mitochondrial (Fragment)(SOLTU:P27081)
chrom_15	19367597	19459040	DRNTG_00823.1	-
chrom_17	3877215	3877734	DRNTG_07493.1	-
chrom_17	3884570	3885375	DRNTG_07491.1	(TrEMBL)Acyl-coenzyme A thioesterase 13 (Fragment)(9ARAE:A0A1D1Y7U4)
chrom_17	3896927	3897383	DRNTG_07490.1	-
chrom_17	3918976	3920856	DRNTG_07489.1	Cytochrome c-type biogenesis CcmH-like mitochondrial protein(ORYSJ:Q6K7S7)
chrom_17	3924485	3925384	DRNTG_07488.1	Cytochrome c-type biogenesis CcmH-like mitochondrial protein(ORYSI:B8AFK5)
chrom_17	3959026	3959941	DRNTG_07487.1	-
chrom_17	3967202	3969537	DRNTG_07486.1	50S ribosomal protein L1, chloroplastic(SPIOL:Q9LE95)
chrom_17	3969570	3969827	DRNTG_07485.1	-
chrom_17	3978228	3979444	DRNTG_07484.1	24-methylenesterol C-methyltransferase 2(ORYSJ:O82427)
chrom_17	3986569	3989639	DRNTG_07482.1	Cytochrome c-type biogenesis CcmH-like mitochondrial protein(ORYSJ:Q6K7S7)
chrom_17	3986754	3989091	DRNTG_07483.1	Cytochrome c-type biogenesis CcmH-like mitochondrial protein(ORYSJ:Q6K7S7)
chrom_17	4026863	4027853	DRNTG_07481.1	Cytochrome c-type biogenesis CcmH-like mitochondrial protein(ORYSI:B8AFK5)
chrom_17	4106911	4108859	DRNTG_01733.1	Non-specific lipid-transfer protein Cw18(HORVU:Q43871)
chrom_17	4108922	4112102	DRNTG_01734.1	Mitochondrial arginine transporter BAC2(ARATH:Q9CA93)
chrom_17	3875895	3883658	DRNTG_07492.1	Putative E3 ubiquitin-protein ligase LIN-1(LOTJA:C6L7U1)
chrom_17	4095647	4096298	DRNTG_01732.1	-
chrom_17	4053722	4106059	DRNTG_01731.1	Protein SWEETIE(ARATH:F4HRS2)
chrom_19	8230520	8231387	DRNTG_01547.1	-
chrom_19	8307448	8308110	DRNTG_01549.1	-
chrom_19	8314683	8319901	DRNTG_01550.1	-
chrom_19	8319680	8322207	DRNTG_01551.1	Glycerol-3-phosphate acyltransferase RAM2(MEDTR:K7PEY4)
chrom_19	8306157	8311914	DRNTG_01548.1	EID1-like F-box protein 3(ARATH:Q93ZT5)
chrom_19	17790629	17791141	DRNTG_03384.1	Mannose-specific lectin(GALNI:P30617)
chrom_19	17801425	17802462	DRNTG_03385.1	Inorganic phosphate transporter 1-11(ORYSJ:Q94DB8)
chrom_19	17850805	17857145	DRNTG_03386.1	(TrEMBL)uncharacterized protein LOC103722397 isoform X1(PHODC:A0A2H3ZB91)
chrom_19	17964831	17971340	DRNTG_03389.1	Remorin 4.1(ORYSJ:Q7XII4)
chrom_19	17858513	17859406	DRNTG_03387.1	-
chrom_19	17914955	17927446	DRNTG_03388.1	Auxin response factor 12(ORYSI:Q258Y5)

Materials and Methods

Table of contents

S1. Reference assembly
S1.1 Whole-genome sequencing using Oxford Nanopore Technology
S1.2 Quality control
S1.3 *De novo* assembly
S1.4 Polishing and removing duplicated contigs
S1.5 Gene prediction and annotation
S2. Generation of pseudo-chromosomes by anchoring contigs onto a linkage map
S2.1 Preparing the mapping population
S2.2 Whole-genome resequencing
S2.3 Quality control and alignment
S2.4 Identification of parental line-specific heterozygous markers
S2.5 Anchoring and ordering contigs
S3. Genetic diversity analysis
S3.1 Whole-genome resequencing of Guinea yam accessions
S3.2 Quality control, alignment, and SNP calling
S3.3 Unsupervised clustering analysis
S3.4 Polymorphism and ploidy of nuclear genomes
S4. Phylogenomic analysis of African yam
S4.1 Data preparation
S4.2 Neighbor-joining tree
S4.3 Inferring the evolutionary history of wild *Dioscorea* species using ∂a∂i
S4.4 Inferring the evolutionary history of wild *Dioscorea* species using fastsimcoal2
S5. Test of hybrid origin
S5.1 Site frequency spectrum polarized by two candidate progenitors of Guinea yam
S5.2 Inferring the domestication history of Guinea yam using ∂a∂i
S5.3 Comparison of F_{ST} on each chromosome among three African yams
S6. Haplotype network analysis of the whole plastid genome
S7. Inferring the changes in population size
S8. Exploring the possibility of extensive introgression from wild *Dioscorea* species

S1. Reference assembly

S1.1 Whole-genome sequencing using Oxford Nanopore Technology

To generate version 2 of the *Dioscorea rotundata* reference genome sequence, we sequenced an F1 individual plant named "TDr96_F1" using the PromethION sequencer (Oxford Nanopore Technologies). "TDr96_F1" was the same individual plant used to obtain version 1 of the *D. rotundata* reference genome sequence (*1*). "TDr96_F1" DNA was extracted from fresh leaves as described (*1*). The DNA was subjected to size selection and purification with a gel extraction kit (Large Fragment DNA Recovery Kit; Zymo Research). The purified DNA was sequenced using PromethION at GeneBay, Yokohama, Japan (http://genebay.co.jp).

S1.2 Quality control

As a first step in our pipeline for genome assembly (Fig. SM1), we removed the lambda phage genome from raw reads with NanoLyse v1.1 (*2*). We then filtered out reads with an average read quality score of less than 7 and those shorter than 1,000 bases with Nanofilt v2.2 (*2*). This was followed by trimming of the first 75 bases to remove low-quality bases in all read that were retained. This generated 3,124,439 reads, corresponding to 20.89 Gbp of sequence (Table SM1).

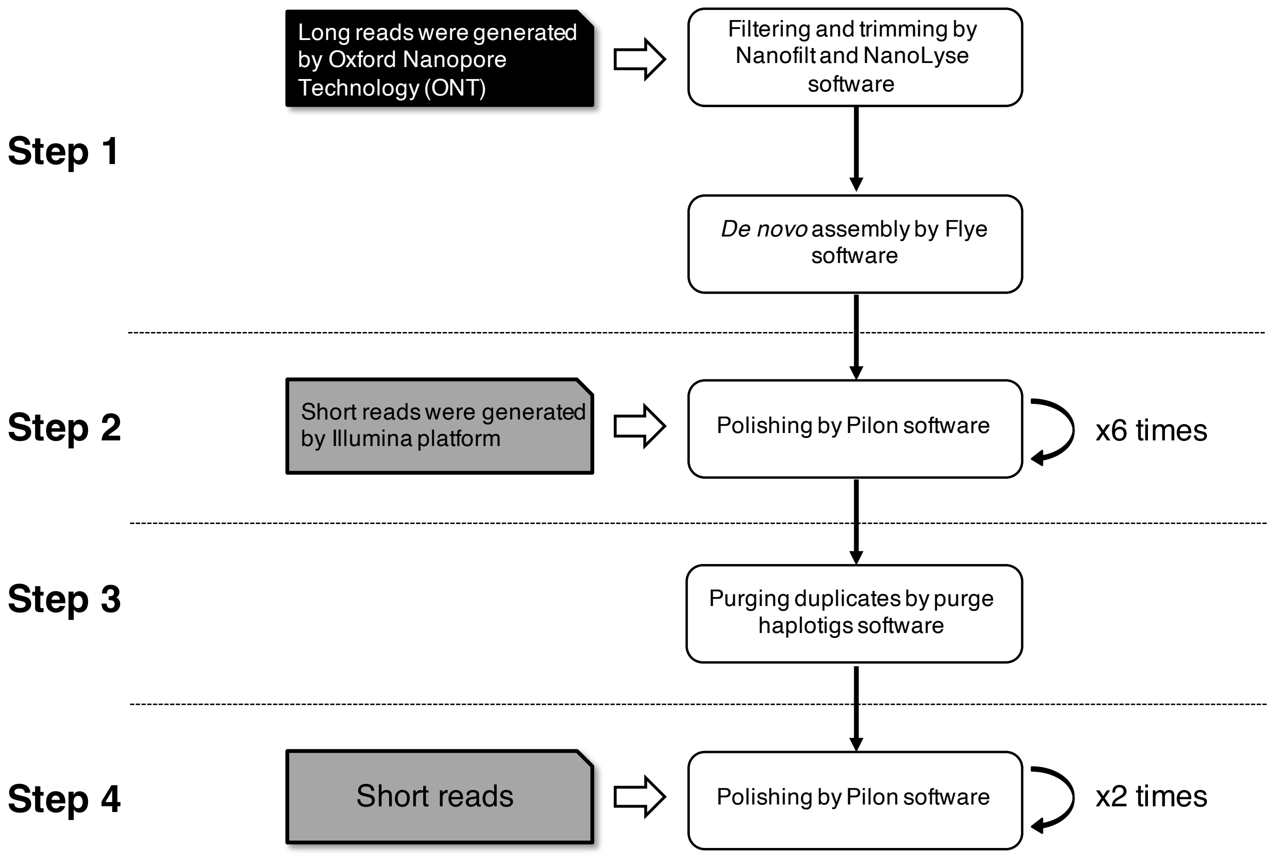

Fig. SM1. Pipeline of genome assembly Ver. 2.

Table SM1. Summary of filtered ONT reads.

Summary	
Number of reads	3,124,439
Total base pairs (Gb)	20.89
Genome coverage	36.6x
Average fragment size (Kb)	6.7
Longest fragment	211,597
Shortest fragment	1,000
Fragment N50 (Kb)	8.0

- Raw reads were registered in DDBJ under accession number DRR196916.
- Genome coverage was estimated based on the expected genome size of *D. rotundata* (570Mb).

S1.3 *De novo* assembly

We assembled filtered long DNA sequence reads with Flye v2.4.2 (*3*), using 570 Mb as the estimated genome size of *D. rotundata* (*1*). This generated 8,721 contigs with N50 of 137,007 base pairs (Step 1 in Table SM2) and a total size of 636.8 Mb, which is larger than the expected *D. rotundata* genome size of 570 Mb. To evaluate the completeness of the gene set in the assembled contigs, we applied BUSCO analysis (Bench-Marking Universal Single Copy) v3.0.2 (*4*). For BUSCO analysis, we set "genome" as the assessment mode and used Embryophyta *odb9* as the database and obtained 40.7% complete BUSCOs (Step 1 in Table SM2).

Table SM2. Summary of the reference assembly.

	Step 1	Step 2	Step 3	**Step 4**
Total number of contigs	8,721	8,721	6,513	**6,513**
Total base-pairs (Mbp)	636.8	628.2	579.7	**579.4**
Average contig size (bp)	73,008	72,029	89,004	**88,961**
Longest contig (bp)	2,301,335	2,267,833	2,267,833	**2,267,326**
Shortest contig (bp)	171	171	171	**171**
N50 (bp)	137,007	134,605	152,963	**152,929**
Complete BUSCOs (%)	40.7	89.9	89.3	**90.1**
Complete and single-copy BUSCOs (%)	39.9	83.9	84.9	**85.7**
Complete and duplicated BUSCOs (%)	0.8	6.0	4.4	**4.4**
Fragmented BUSCOs (%)	8.2	3.2	3.2	**3.1**
Missing BUSCOs (%)	51.1	6.9	7.5	**6.8**

S1.4 Polishing and removing duplicated contigs

To correct the assembled contigs, we repeatedly polished them with Illumina short reads (Table SM3) using Pilon v1.23 (*5*) until there was no further change in the % of complete BUSCOs. We aligned Illumina jump reads as single reads to the assembled contigs using the bwa mem command in BWA v0.7.17 (*6*) and sorted the BAM files with SAMtools v1.9 (*7*). The BAM files were used to run Pilon with the option "--diploid". We polished the contigs six times. The percentage of complete BUSCOs

was 89.9% after the first polishing step (Step 2 in Fig. SM1). To remove duplicated contigs, we used Purge Haplotigs v1.0.2 (*8*), which removes duplicated contigs based on depth and the number of matching bases (Step 3 in Fig. SM1). In Purge Haplotigs, the percent cutoff of aliment coverage was set to 95%. Finally, we polished the contigs again. The percentage of complete BUSCOs was 90.1% after the second polishing process (Step 4 in Fig. SM1). Comparing the features in the old reference genome with the new reference genome, the number of missing bases ("N") was drastically reduced (Table SM4).

Table SM3. Sequence list used for polishing.

Name	Sequence Platform	Total size (Gb)	Genome coverage	Accession No.
Fragment (PE)	Illumina Miseq	16.77	29.4x	DRR027644
MP jump reads (as Single)				
for 2k	Illumina Hiseq 2500	6.43	11.3x	DRR027645
for 3k	Illumina Hiseq 2500	7.56	13.3x	DRR027646
for 4k	Illumina Hiseq 2500	6.18	10.8x	DRR027647
for 5k	Illumina Hiseq 2500	7.20	12.6x	DRR027648
for 6k	Illumina Hiseq 2500	7.27	12.8x	DRR027649
for 8k	Illumina Hiseq 2500	6.79	11.9x	DRR027650

- All values are calculated after quality control.
- Genome coverage was estimated based on the expected genome size of *D. rotundata* (570 Mb).

Table SM4. Comparison of the old (*1*) and new reference assemblies.

Feature	Ver. 1	Ver. 2
Number of scaffolds*	4,723	6,513
Total scaffold* size (Mbp)	594.23	579.41
Longest scaffold* (Mbp)	13.61	2.28
N50 (Mbp)	2.12	0.15
Total 'N' bp	90,097,902	953
Complete BUSCOs (%)	90.7	90.1

*In Version 2, contigs were used instead of scaffolds.

S1.5 Gene prediction and annotation

For gene prediction, we used 20 RNA-Seq data sets representing 15 different organs and three different flowering stages in male and female plants (Table SM5). Total RNA was used to construct cDNA libraries using a TruSeq RNA Sample Prep Kit V2 (Illumina) according to the manufacturer's instructions. The extracted RNA was sequenced on the Illumina platforms NextSeq500 and HiSeq4000. In the quality control step, we filtered the reads and discarded reads shorter than 50 bases and those with an average read quality below 20 and trimmed poly(A) sequences with FaQCs v2.08 (*9*). Quality trimmed reads were aligned to the newly assembled contigs with HISAT2 v2.1 (*10*) with the options "--no-mixed --no-discordant --dta". Transcript alignments were assembled with StringTie v1.3.6 (*11*) separately for each BAM file. These GFF files were

integrated with TACO v0.7.3 (*12*) with the option "--filter-min-length 150", generating 26,609 gene models within the new assembly (Table SM6). Additionally, coding sequences (CDSs) that were predicted using the previous reference genome (*1*) were aligned to the newly assembled contigs with Spaln2 v2.3.3 (*13*). Consequently, 8,889 CDSs that did not overlap with the new gene models were added to the new gene models (Table SM6). Finally, gene models shorter than 75 bases were removed, and InterProScan v5.36 (*14*) was used to predict ORFs (open reading frames) and strand information for each gene model. We predicted 35,498 genes, including 66,561 transcript variants (Table SM6). For gene annotation, the predicted gene models were searched in the Pfam protein family database using InterProScan (*14*) and with the blastx command in BLAST+ (*15*) with the option "-evalue 1e-10", using the Viridiplantae database from UniProt as the target database. The resulting gene models and annotations were uploaded to ENSEMBL (http://plants.ensembl.org/Dioscorea_rotundata/Info/Index).

Table SM5. Summary of RNA-seq data used for gene prediction.

Sample name	Fastq size		Sequence platform	Comment	Accession No.
	Original (Gbp)	Filtered (Gbp)			
01_Flowers-rachis-top	4.36	4.28	NextSeq500	Top 2 cm of inflorescence	DRR063119
02_Flowers-rachis-lower	4.96	4.87	NextSeq500	Lower 2 cm of inflorescence	DRR063118
03_Flower-bud	3.52	3.46	NextSeq500	Flower bud	DRR063116
04_Axillary-bud	4.31	4.23	NextSeq500	Axillary bud	DRR063115
05_Leaf	3.26	3.18	NextSeq500	Leaf	DRR045127
06_Petiole	4.47	4.38	NextSeq500	Petiole	DRR063121
07_Pulvinus	4.66	4.58	NextSeq500	Pulvinus	DRR063120
08_Rachis	4.59	4.51	NextSeq500	Rachis	DRR063117
09_Stem	3.45	3.36	NextSeq500	Young_stem	DRR045129
10_Spine	4.51	4.43	NextSeq500	Spine	DRR063123
11_Root	3.62	3.54	NextSeq500	Root	DRR063122
12_Tuber-head	4.72	4.65	NextSeq500	Tuber (head)	DRR063126
13_Tuber-middle	4.06	4.00	NextSeq500	Tuber (middle)	DRR063125
14_Tuber-tail	4.48	4.40	NextSeq500	Tuber (tail)	DRR063124
15_fem_Y917-1	4.12	4.08	HiSeq4000	TDr97_00917 female flower early stage 1	DRR208398
16_fem_Y917-2	4.27	4.23	HiSeq4000	TDr97_00917 female flower early stage 2	DRR208399
17_fem_Y917-3	4.43	4.37	HiSeq4000	TDr97_00917 female flower early stage 3	DRR208400
18_mal_Y777-1	4.48	4.42	HiSeq4000	TDr97_00777 male flower early stage 1	DRR208401
19_mal_Y777-2	3.43	3.40	HiSeq4000	TDr97_00777 male flower early stage 2	DRR208402
20_mal_Y777-3	4.13	4.09	HiSeq4000	TDr97_00777 male flower early stage 3	DRR208403

Table SM6. Summary of gene prediction.

	Contigs (6,513)	Pseudo Chrom. (01~20)
No. genes	35,498	30,344
(Total transcript variants)	(66,561)	(57,637)
ORF status		
Complete	22,423	19,502
5′ partial	1,225	1,018
3′ partial	10,385	8,594
Internal	559	465
No ORF	906	765
Prediction software		
TACO (*12*)	26,609	23,335
Spaln2 (*13*)	8,889	7,009

S2. Generation of pseudo-chromosomes by anchoring contigs onto a linkage map

S2.1 Preparing the mapping population

To develop the chromosome-scale TDr96_F1 genome sequence from the assembled contigs, we generated an F1 population containing 156 individuals by crossing two *D. rotundata* breeding lines: TDr04/219 as the female parent (P1) and TDr97/777 as the male parent (P2).

S2.2 Whole-genome resequencing

We extracted each DNA sample from dried *D. rotundata* leaves as described (*1*). Libraries for PE short reads were constructed using an Illumina TruSeq DNA LT Sample Prep Kit (Illumina). The PE library was sequenced on the Illumina Hiseq4000 platform. A summary of sequence and alignment information is provided in Table SM7 (attached at the bottom of this file).

S2.3 Quality control and alignment

We used FaQCs v2.08 (*9*) to remove unpaired reads and adapters. We then filtered out reads shorter than 75 bases or those whose average read quality score was 20 or lower with prinseq-lite v0.20.4 lite (*16*). We also trimmed bases whose average read quality score was below 20 from the 5′ end and the 3′ end using the sliding window approach (the window size was five bases, and the step size was one base) in prinseq-lite (*16*). Subsequently, we aligned the filtered reads of P1, P2, and F1 progenies to the newly assembled contigs (supplementary text S1) using the bwa mem command in BWA (*6*). After sorting the BAM files, we only retained properly paired and uniquely mapped reads using SAMtools (*7*).

S2.4 Identification of parental line-specific heterozygous markers

SNP-type heterozygous markers

SNP-based genotypes for P1, P2, and F1 progenies were obtained as a VCF file. The VCF file was generated as follows: (i) SAMtools v1.5 (*7*) mpileup command with the option "-t DP,AD,SP -B -Q 18 -C 50"; (ii) BCFtools v1.5 (*17*) call command with the option "-P 0 -v -m -f GQ,GP"; (iii)

BCFtools (*17*) view command with the options “-i 'INFO/MQ≥40, INFO/MQ0F≤0.1, and AVG(GQ)≥10”; and (iv) BCFtools (*17*) norm command with the option “-m+any” (Fig. SM2). We rejected the variants with low read depth (<10) or low genotype quality scores (<10) in the two parents. We regarded variants with low read depth (<8) or low genotype quality scores (<5) in F1 progenies as missing and only retained the variants with low missing rates (<0.3).

Subsequently, only bi-allelic SNPs were selected by the BCFtools (*17*) view command with the option “-m 2 -M 2 -v snps”. Referring to the genotypes in the VCF file, heterozygous genotypes called by unbalanced allele frequency (out of 0.4-0.6 in two parents, and out of 0.2-0.8 in F1 progenies) were regarded as missing, and filtering for missing rate (<0.3) was applied again. Finally, a binomial test was performed to reject SNPs affected by segregating distortion in the F1 progenies. This binomial test assumes that the probability of success rate is 0.5 based on the two-side hypothesis, and we regarded variants having *p*-value less than 0.2 as segregation distortion.

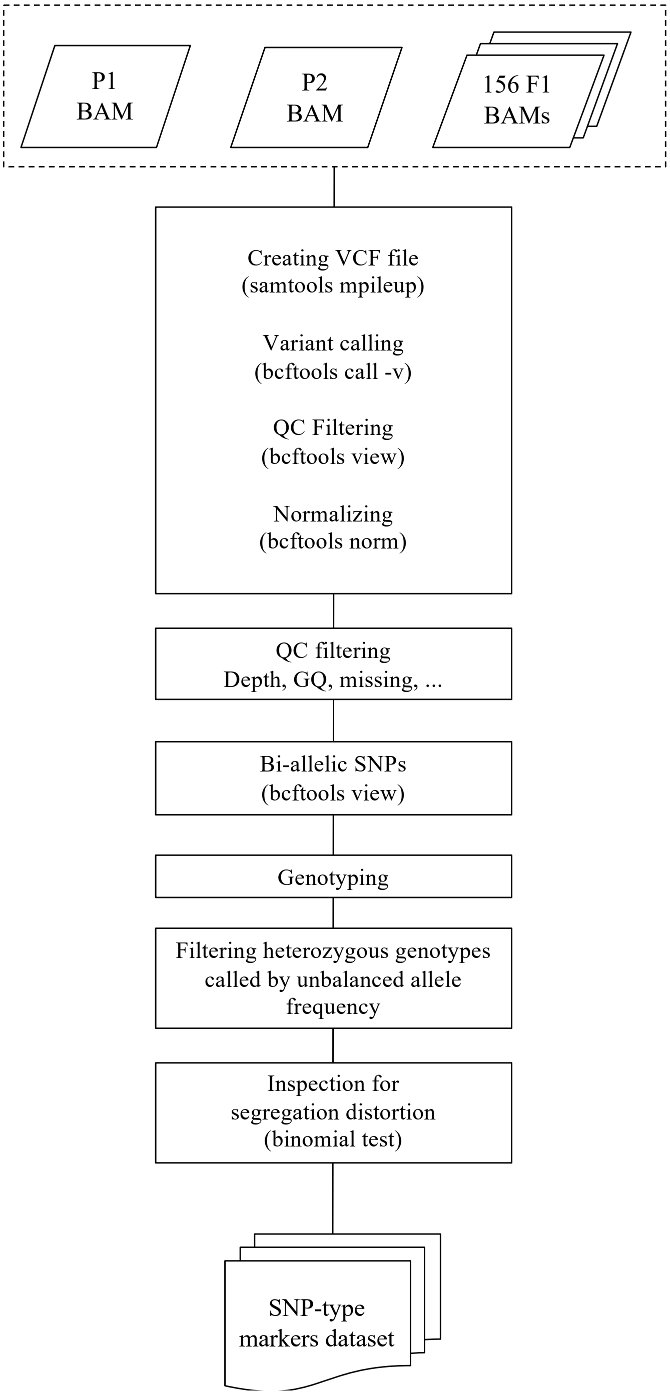

Fig. SM2. Flowchart of SNP-type heterozygous marker selection.

Presence/absence-type heterozygous markers.
A VCF file was generated to search for positions with contrasting read depth between the two parental plants P1 and P2 using the following commands: (i) SAMtools (*7*) mpileup command with the option "-B -Q 18 -C 50"; (ii) BCFtools (*17*) call command with the option "-A"; and (iii) BCFtools (*17*) view command with the options "-i 'MAX(FMT/DP)≥8 and MIN(FMT/DP)≤0' -g miss -V indels". This means that one of the parents (P1 or P2) has enough read depth (≥8) and another parent has no reads aligned on that region (A in Fig. SM3). Subsequently, we converted continuous positions in the VCF file to a feature that provides the start and end coordinate information of a region using the BEDTools v.2.26 (*18*) merge command with the option "-d 10 -c 1 -o count". We only retained sufficiently wide features (≥50 bp) in the BED file (the 1st BED). To reject false positives whereby low-depth regions are erroneously regarded as absent regions, we focused on both the boundary regions around each feature and the features themselves. For boundary regions, the 2nd BED file including expanded (twice-sized) features of each feature given in the 1st BED was generated with the BEDTools (*18*) slop command with the option "-b 0.5 –pct".

Using the depth value in each feature given in the 1st BED, presence/absence-based genotypes for parental plants P1 and P2 and F1 progenies were determined. To verify the rejection of false-positive features, we also referred to the depth values in the boundary regions around each feature. Verified features were only accepted as presence/absence markers. The depth values in each feature were calculated with the SAMtools (*7*) bedcov command with the option "-Q 0". Also, the depth values in the boundary regions were obtained by subtracting the depth values of the 2nd BED from that of the 1st BED (B in Fig. SM3). For P1 and P2, we regarded genotypes having depth ≥ 8 as present genotypes, meaning the heterozygosity of present and absent, while those having depth < 2 were classified as absent genotypes, meaning the homozygosity of absent. For F1 progenies, we classified markers with depth > 0 and = 0 as present and absent markers, respectively. Finally, we applied the same binomial test for SNP-type heterozygous markers as that used for presence/absence-type heterozygous markers.

A

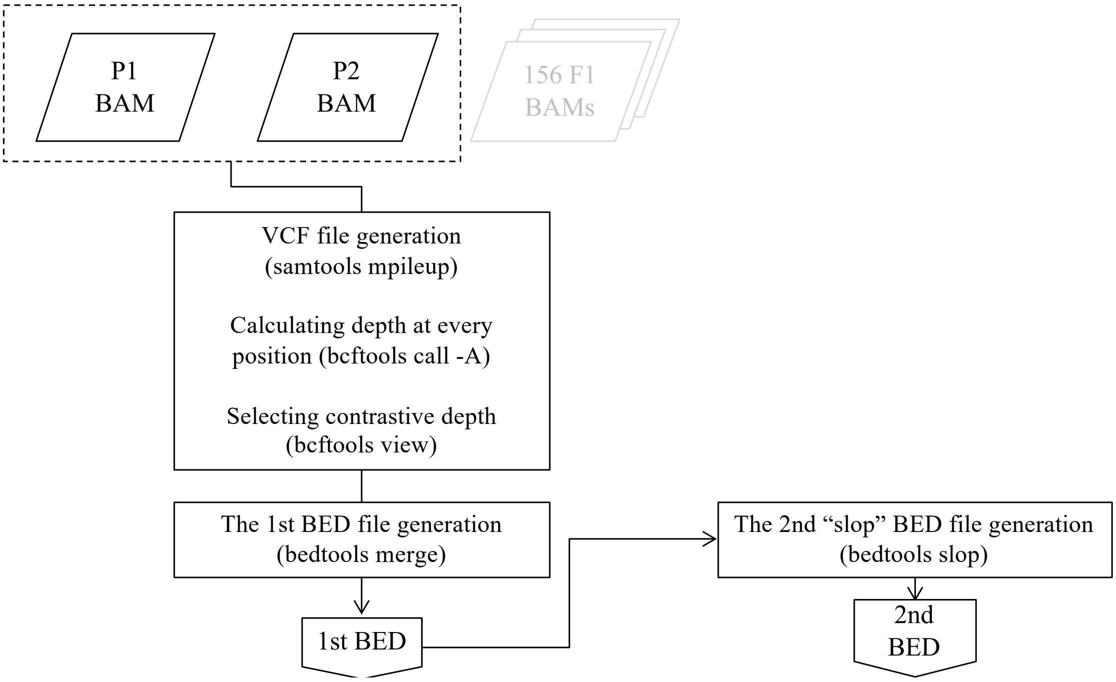

(continued)

B

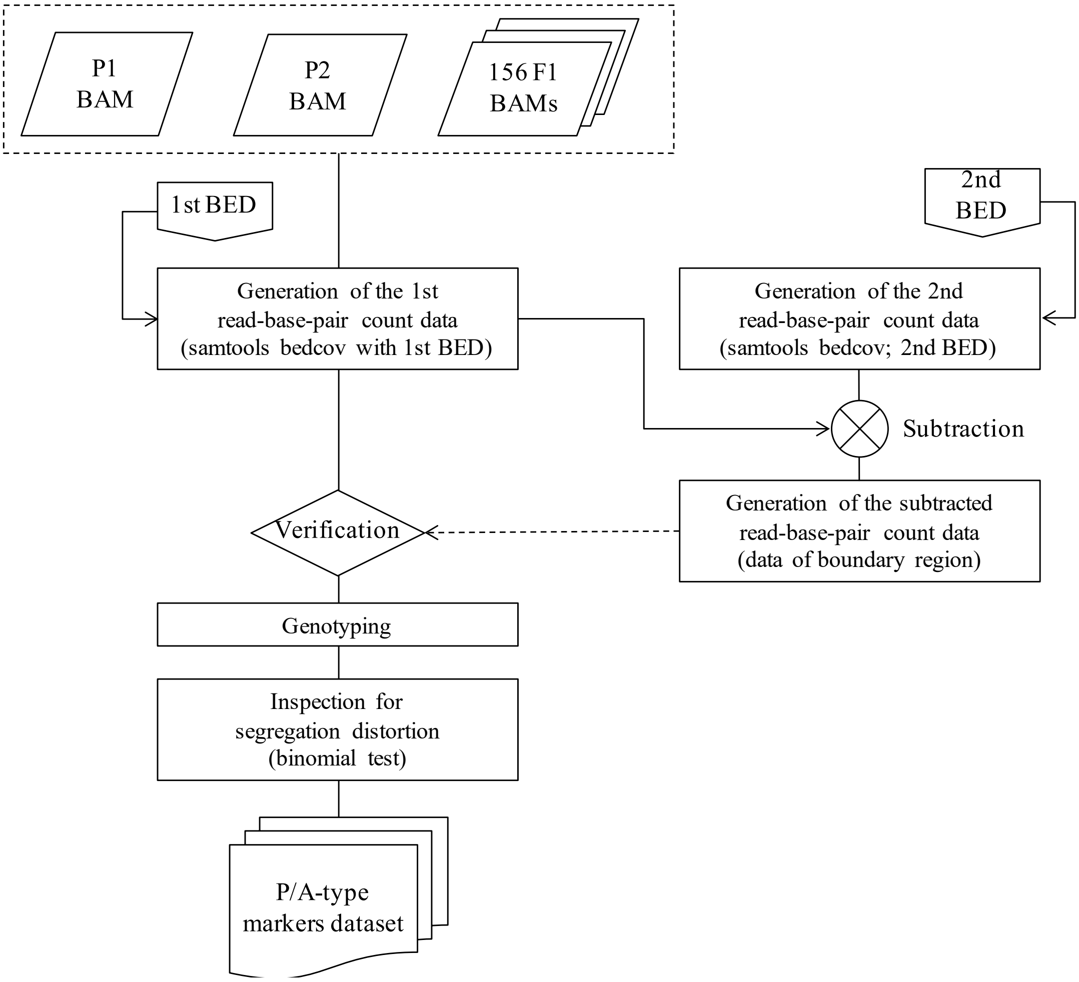

Fig. SM3. Flowchart of presence/absence-type heterozygous marker selection.

Integration of SNP-type and presence/absence-type heterozygous markers. To develop parental line-specific linkage maps, we integrated SNP-type and P/A-type (presence/absence-type) heterozygous markers. Two types of markers were defined: Type-1 markers and Type-2 markers. If an SNP-type marker was heterozygous in P1 but homozygous in P2 or if a P/A-type marker was present in P1 and absent in P2, it was classified as a Type-1 marker (P1-heterozygous marker set). Conversely, if a SNP-type marker was homozygous and heterozygous in P1 and P2, respectively, or if a P/A-type marker was absent in P1 but present in P2, it was classified as a Type-2 marker (P2-heterozygous marker set).

S2.5 Anchoring and ordering contigs

Pruning and flanking markers based on Spearman's correlation coefficients. Distance matrices of Spearman's correlation coefficients (ρ) were calculated for every marker pair in each contig in each marker set (P1-heterozygous marker set and P2-heterozygous marker set). According to the histogram of absolute ρ calculated from each contig, most markers on the same contigs were correlated with each other (Fig. SM4). Therefore, we pruned correlated flanking markers to remove redundant markers (Fig. SM5). Accordingly, we obtained 11,389 markers for linkage mapping (Table SM8).

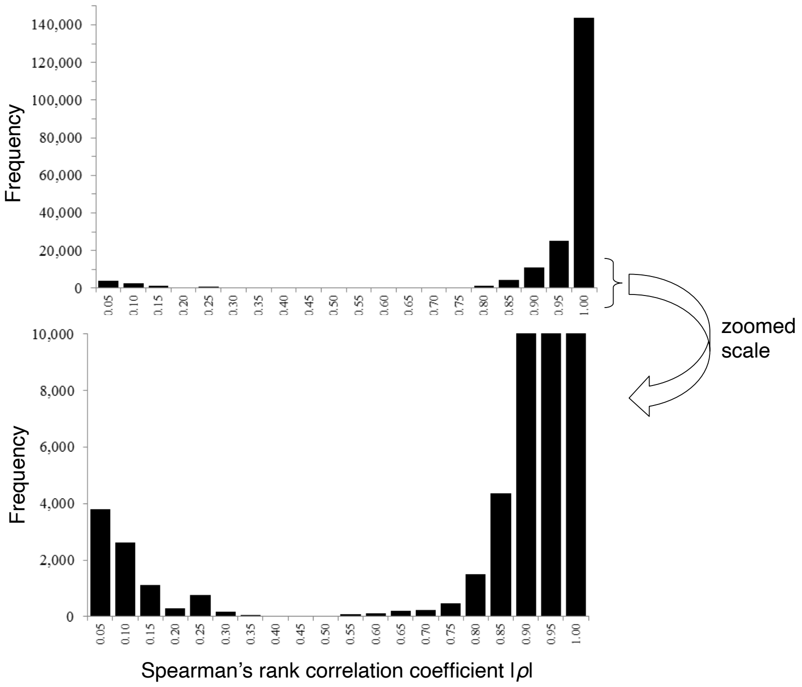

Fig. SM4. Histogram of absolute ρ values calculated from each contig.

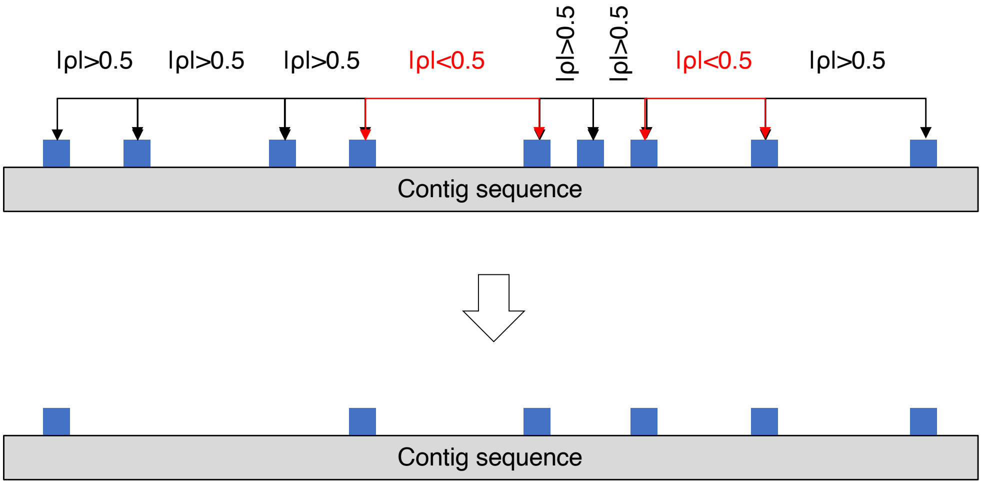

Fig. SM5. The process used to prune correlated flanking markers.

Table SM8. Summary of the anchoring markers.

	Type1	Type2	Type1 + Type2
**Total anchoring markers to generate linkage groups**	7,020	4,369	11,389
- SNP	4,607	3,435	8,042
- P/A	2,413	934	3,347
Total base pairs of linkage group having markers (Mbp)	434.7	328.4	495.2
Total anchored base pairs estimated from genome size (%)	75.5	56.7	85.5

Linkage mapping. The markers obtained as described in the previous section were converted to genotype-formatted data. Based on this genotype-formatted data, genetic linkage maps were constructed using MSTmap (*19*) with the following parameters: "population_type DH; distance_function kosambi; cut_off_p_value 0.000000000001; no_map_dist 15.0; no_map_size 0; missing_threshold 25.0; estimation_before_clustering no; detect_bad_data no; objective_function ML" for each marker set. After trimming the orphan linkage groups, we solved the complemented-phased duplex linkage groups caused by coupling-type and repulsion-type markers in the pseudo-testcross method. Finally, two parental-specific linkage maps were constructed. These two linkage maps were designated as P1-map (constructed using Type-1 markers) and P2-map (constructed using Type-2 markers) (Fig SM6 and Fig SM7). The linkage groups were visualized by R/qtl (*20*). The numbering of linkage groups is the same as that used in the previous reference genome (*1*).

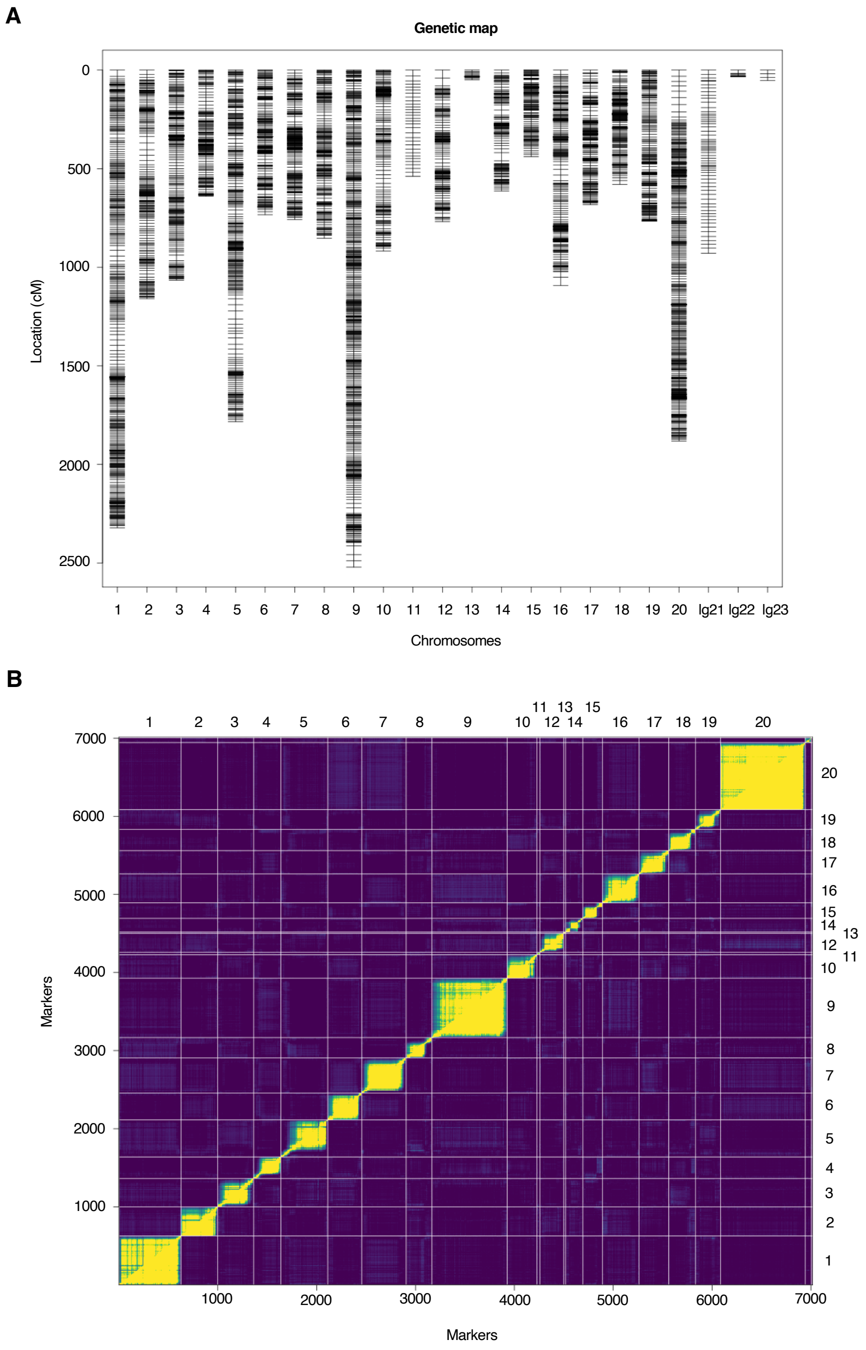

Fig. SM6. P1-map created using P1 heterozygous markers. (A) Contig positions in the P1-map. (B) Estimated recombination fractions (upper-left triangle) against LOD score (lower-right triangle) plotted by R/qtl (*20*).

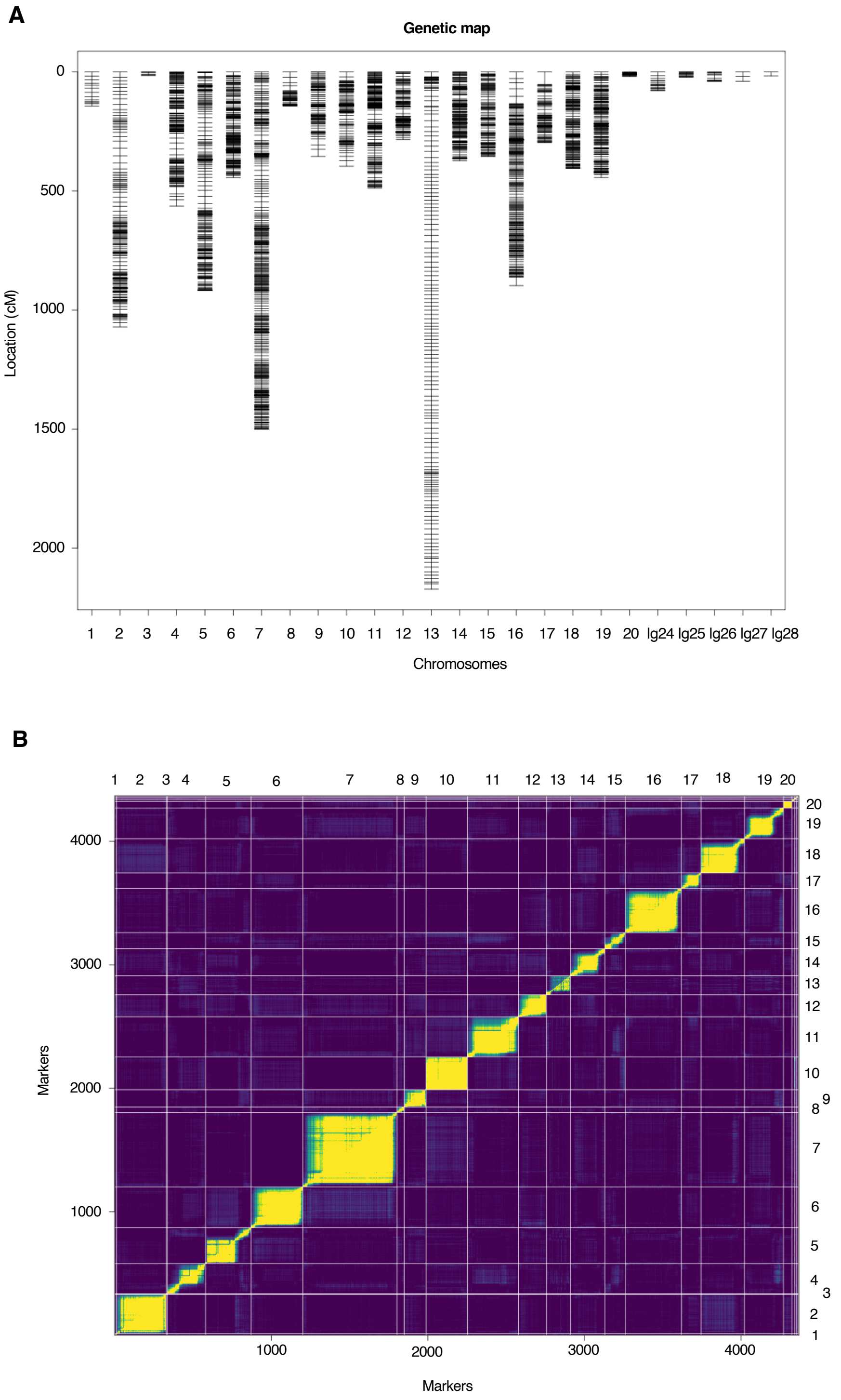

Fig. SM7. P2-map created using P2 heterozygous markers. (A) Contig positions in the P2-map. (B) Estimated recombination fractions (upper-left triangle) against LOD score (lower-right triangle) plotted by R/qtl (*20*).

Integration of two parental-specific linkage maps into the chromosome-scale physical genome sequence. Based on a matrix derived from the contigs shared between the P1- and P2-maps, i.e., linkage groups (Table SM9), the contigs were anchored and linearly ordered as pseudo-chromosomes. During the anchoring and ordering process, we identified contigs whose

markers were allocated to different linkage groups. Such contigs were further divided into sub-contigs to ensure that they were not allocated to different pseudo-chromosomes. We divided the contigs at the proper positions as described previously (*1*). During this procedure, 34 genes including 61 transcript variants were cut and removed. Finally, a previously described method (*1*) was followed to generate the pseudo physical genome sequence composed of 20 pseudo-chromosomes. To compare the newly generated pseudo-chromosomes with the ones we constructed previously (*1*), we generated a dot plot with D-Genies (*21*) (Fig. SM8) and counted the anchored base pairs in the new pseudo-chromosomes (Table SM10). The resulting reference genome, including unanchored contigs, was uploaded to ENSEMBL (http://plants.ensembl.org/Dioscorea_rotundata/Info/Index).

Table SM9. A matrix of the number of shared contigs between the P1-map and P2-map. Linkage groups (lg) 21-28 do not have shared contigs.

		P2-map																								
		1	2	3	4	5	6	7	8	9	10	11	12	13	14	15	16	17	18	19	20	lg24	lg25	lg26	lg27	lg28
P1-map	1	5	2	1	2	0	3	2	0	0	3	2	1	0	1	0	5	0	2	0	1	0	0	0	0	0
	2	0	120	0	1	2	2	3	0	1	1	1	0	0	0	0	1	0	1	2	0	0	0	0	0	0
	3	0	2	3	1	0	3	9	0	1	0	0	0	0	0	0	1	0	1	2	0	0	0	0	2	2
	4	0	0	0	84	2	0	1	0	0	0	0	0	0	3	0	1	0	0	0	0	0	0	0	0	0
	5	0	1	0	3	135	2	3	0	1	1	2	2	0	4	1	0	1	1	2	0	0	0	0	0	0
	6	0	0	0	0	3	128	2	0	1	1	2	0	0	1	0	2	0	0	2	0	0	0	0	0	0
	7	0	2	0	1	2	2	199	0	1	1	3	0	0	0	1	1	0	0	3	0	0	0	0	0	0
	8	0	0	0	1	1	4	1	24	0	0	0	0	0	0	1	4	1	2	1	0	9	0	0	0	0
	9	0	1	0	0	2	4	4	0	71	4	1	0	0	2	1	5	1	0	1	0	0	0	0	0	0
	10	0	1	0	0	0	0	1	0	0	93	1	1	0	1	1	0	1	0	0	0	0	6	0	0	0
	11	0	0	0	0	0	0	1	0	0	0	8	0	0	0	0	0	0	1	0	0	0	0	0	0	0
	12	0	0	0	0	2	0	1	0	0	2	2	75	1	0	1	2	0	5	0	0	0	0	0	0	0
	13	0	0	0	0	0	0	1	0	0	0	0	0	5	0	0	0	0	0	0	0	0	0	0	0	0
	14	0	0	0	2	1	1	1	0	0	2	0	0	1	66	0	0	1	0	1	1	0	0	0	0	0
	15	0	0	0	0	0	0	0	0	1	0	1	0	0	2	42	2	0	0	1	0	0	0	0	0	0
	16	0	1	0	0	2	0	2	0	2	0	1	1	0	0	0	126	1	1	0	0	0	0	0	0	0
	17	0	0	0	1	2	1	1	0	0	0	1	0	1	1	1	2	60	0	0	0	0	0	0	0	0
	18	0	1	0	0	0	2	1	0	0	1	2	1	0	0	0	0	0	118	0	0	0	0	0	0	0
	19	0	1	0	0	0	1	2	0	0	0	2	0	4	0	0	0	0	1	100	0	0	0	0	0	0
	20	1	8	0	0	5	1	4	0	0	5	6	2	3	2	0	4	1	1	0	39	0	0	3	0	0
	lg21	0	0	0	0	0	0	1	0	0	0	0	0	1	0	0	0	0	0	0	0	0	0	0	0	0
	lg22	0	0	0	0	0	0	0	0	0	0	6	0	0	0	0	0	0	0	0	0	0	0	0	0	0
	lg23	0	0	0	0	0	0	0	0	0	0	1	0	0	0	0	0	0	0	0	0	0	0	0	0	0

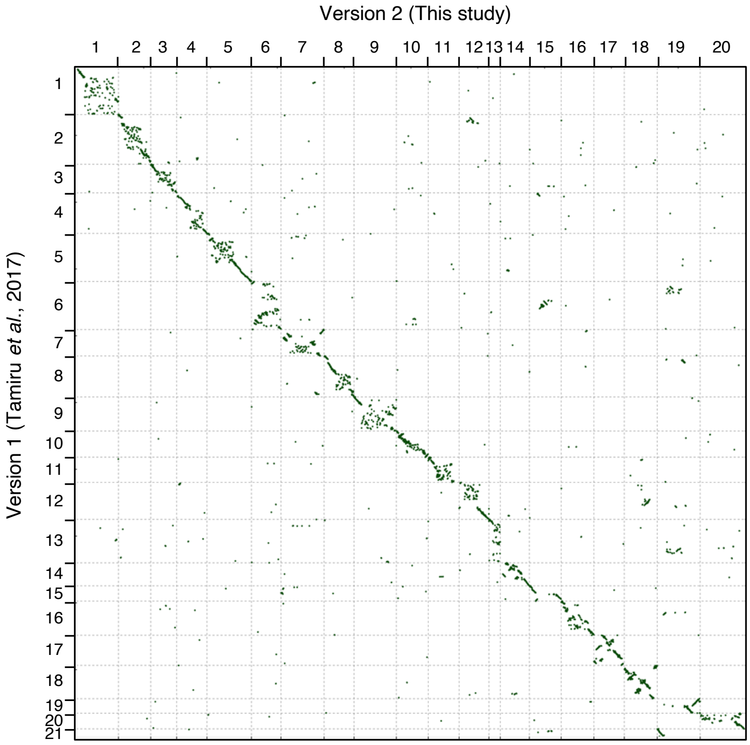

Fig. SM8. Dot plot of the new pseudo-chromosomes (Ver. 2) against the previously generated pseudo-chromosomes (Ver. 1) (*1*).

Table SM10. Comparison of the old (Ver. 1) (*1*) and new (Ver. 2) pseudo-chromosomes.

Feature	Ver. 1	Ver. 2
Number of Pseudo Chr.	21	20
Total size of Pseudo Chr. (Mbp)	456.67	491.97
Total not 'N' Mbp	406.1	487.31
Total size of Pseudo Chr. / Total scaffold* (%)	76.9	84.9
Complete BUSCOs (%)	82.8%	82.3%

*In version2, contigs were used instead of scaffolds.

S3. Genetic diversity analysis

S3.1 Whole-genome resequencing of Guinea yam accessions

For genetic diversity analysis, we selected 333 accessions of *D. rotundata* maintained at IITA, Nigeria, representing the genetic diversity of Guinea yam landraces and improved lines of West Africa. We extracted DNA from dried leaves of each *D. rotundata* accession as described (*1*). Libraries for PE short reads were constructed using an Illumina TruSeq DNA LT Sample Prep Kit (Illumina). The PE library was sequenced on the Illumina Nextseq500 or Hiseq4000 platform. Finally, P1 (TDr04/219) and P2 (TDr97/777) parents used to anchor the contigs and the reference individual "TDr96_F1" were added to the 333 accessions. Therefore, we used a total of 336 accessions for this analysis. A summary of the sequences and alignments is provided in Dataset S1.

S3.2 Quality control, alignment, and SNP calling

We used FaQCs v2.08 (*9*) and prinseq-lite v0.20.4 lite (*16*) for quality control. We used the same parameters provided in supplementary text S2.3, but both paired and unpaired reads were aligned to the new reference genome using the bwa mem command in BWA (*6*) with option "-a". After sorting the BAM files, the VCF file was generated using the SAMtools (*7*) mpileup command with the option "-t DP,AD,SP -B -Q 18 -C 50", and variants were called by the BCFtools (*17*) call command with the option "-P 0 -v -m -f GQ,GP". Low-quality variants were rejected using the BCFtools (*17*) view command with the options "-i 'INFO/MQ≥40, INFO/MQ0F≤0.1, and AVG(GQ)≥5'". We regarded variants with low read depth (<8) or low genotype quality score (<5) as missing, filtered out SNPs with high missing rates (≥0.3) across all samples, and only retained bi-allelic SNPs on the pseudo-chromosomes.

S3.3 Unsupervised clustering analysis

Through the pipeline described in supplementary text S3.2, 6,124,093 SNPs were retained in 336 Guinea yam accessions. The VCF file including 336 Guinea yam accessions was converted into a GDS file with the gdsfmt v1.20 R package implemented in the SNPRelate v1.18 (*22*) R package. We then ran SNPRelate (*22*) without filtering for principal component analysis (PCA). Moreover, we used sNMF v1.2 (*23*) for admixture analysis of the 336 Guinea yam accessions. To choose the best *K* value, we launched sNMF (*23*) for each *K* value from 2 to 20 (Fig. SM9). We could not find the best *K* value based on the cross-entropy criterion, so we defined five clusters for convenience.

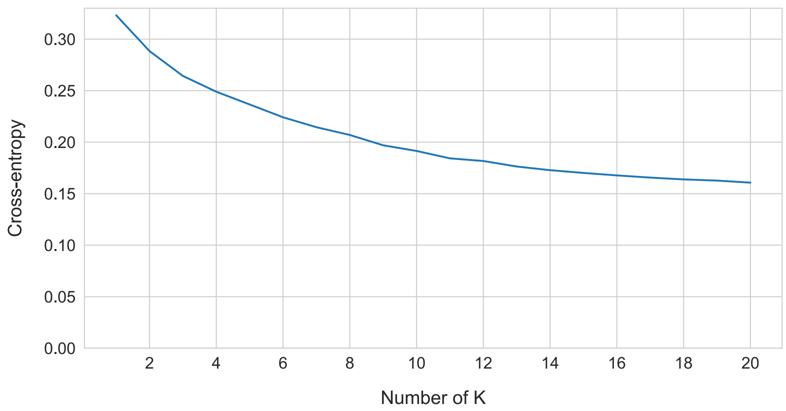

Fig. SM9. Cross-entropy values from *K*=1 to *K*=20 for admixture analysis.

S3.4 Polymorphism and ploidy of nuclear genomes

Heterozygosity level and unique alleles. First, we calculated the heterozygosity level in each accession (Fig. S2). We defined the heterozygosity level as follows:

$$(Heterozygosity\ level) = \frac{S}{L}$$

where S is the number of heterozygous SNPs and L is the total number of mapped sites in an accession. The heterozygosity levels of each cluster were statistically compared by two-tailed Student t test (Table S2). Second, we counted the unique alleles in each cluster (Fig. S3). An allele was considered unique if it only existed in a cluster even when the allele was a singleton in all accessions.

Flow cytometry.
Ploidy level was estimated by flow cytometry using a Partec Ploidy Analyzer (Sysmex Partec, Gorlitz, Germany). Fully developed fresh young leaves were sampled and chopped with a razor blade (ca. 5 x 5 mm) in 0.4 mL nuclear extraction buffer (solution A of a High-resolution kit; Sysmex Partec, Gorlitz, Germany). The suspension was filtered through a nylon filter (50-µm mesh), and the extracted nuclei were stained with 4′,6-diamino-2-phenylindole solution. After 5 min of incubation at room temperature, the sample was examined in a ploidy analyzer at a rate of 5–20 nuclei/s. The DNA index (DI) of each accession was calculated based on the relative amount of DNA in nuclei at the G1 stage compared to the internal standard. Rice (*Oryza sativa* L.) was used as an internal standard for calibration of the measurements. Flow cytometry was repeated two or three times with different leaf samples to confirm the DI of each accession. The ploidy levels of each accession were determined by comparing their DI with that of the diploid accession "TDr1673", for which the chromosome number was confirmed microscopically to be 2n = 40. (Dataset S1)

Summary statistics of population genetics. After removing the triploid accessions of cluster 1, we imputed missing genotypes using BEAGLE v4.1 (*24*) with default options. We then calculated the summary statistics of population genetics (Table S3). First, we counted segregating sites and singletons in 308 Guinea yam accessions. We also estimated Watterson's θ ($\hat{\theta}_W$) (*25*), pairwise nucleotide diversity ($\hat{\theta}_\pi$) (*26*), and Tajima's D (*27*) in the same dataset. We defined $\hat{\theta}_W$ as follows:

$$\hat{\theta}_W = \frac{S}{a * \bar{L}}$$

where a is equal to:

$$a = \sum_{i=1}^{n-1} \frac{1}{i}$$

and $\bar{L}$ is the number of average mapped sites in a population and n is the number of sequences. We also defined $\hat{\theta}_\pi$ as:

$$\hat{\theta}_\pi = \frac{1}{\bar{L}} \frac{n}{n-1} \frac{\sum_{i<j} k_{ij}}{n(n-1)/2}$$

where $\bar{L}$ is the number of average mapped sites in a population, n is the number of sequences, and k_{ij} is the number of nucleotide differences between the ith and jth sequences.

We also calculated LD decay of 308 Guinea yam accessions (Fig. S4). The SNPs whose minor allele frequencies less than 0.05 were removed from the above SNP set used to calculate θ. LD decay was calculated with 200-kb window and 100-kb step. Ten SNPs were randomly sampled within a window, and all possible combinations of r^2 were calculated using the sampled SNPs within a window.

S4. Phylogenomic analysis of African yam

S4.1 Data preparation

For phylogenomic analysis of African yam, we used 308 Guinea yam accessions sequenced in the present study (excluding cluster 1 triploid accessions), as well as 80 *D. rotundata*, 29 *D. abyssinica*, 21 Western *D. praehensilis*, and 18 Cameroonian *D. praehensilis* accessions that were sequenced in a previous study (*28*) using two accessions of the Asian species *D. alata* as an outgroup (Table SM11). Of the samples sequenced in the previous study (*28*), we only used sequences whose species labels matched a species predicted by admixture analysis in the previous study (*28*). Also, we removed the sequences that were labeled as hybrids in the previous study *(28*). Two sequences of *D. alata* downloaded from NCBI were used as the outgroup (Table SM11). Subsequently, read quality control, alignment, and SNP calling of these 458 sequences were conducted using the pipeline described in supplementary text S3.2. Except for the Neighbor-joining (NJ) tree (*29*) (supplementary text S4.2), we only used SNPs with a missing rate < 0.3 in each targeted species. When the markers were polarized by comparison with the *D. alata* outgroup, the SNPs at positions where the alleles of *D. alata* were not completely fixed or where either of the *D. alata* sequences was missing were filtered out.

S4.2 Neighbor-joining tree

Before constructing the NJ tree (*29*), we only retained SNPs at positions with no missing data across all five species (*D. rotundata*, *D. abyssinica*, Western *D. praehensilis*, Cameroonian *D. praehensilis*, and *D. alata*). When we converted the VCF file including the remaining SNPs to a multi-FASTA file, heterozygous SNPs were converted to IUPAC code to characterize them as ambiguous markers. To construct the NJ tree (*29*), we ran MEGA X v10.1.8 (*30*) using the 463,293 remaining SNPs. In MEGA X (*30*), the bootstrap value was set to 100 and the other parameters were set as default. Finally, the NJ tree was drawn with GGTREE v2.0.4 (*31*).

S4.3 Inferring the evolutionary history of wild *Dioscorea* species using ∂a∂i

To elucidate the evolutionary relationships of the three wild *Dioscorea* species, *D. abyssinica* (indicated as A), Western *D. praehensilis* (P), and Cameroonian *D. praehensilis* (C), which are closely related to *D. rotundata*, we performed ∂a∂i analysis (*32*). This technique allows evolutionary parameters to be estimated based on an unfolded site frequency spectrum. The joint unfolded site frequency spectrum was calculated based on the 17,532 polarized SNPs and was projected down to 25 chromosomes in each species.

First, three phylogenetic models, {{A, P}, C}, {{P, C}, A}, and {{C, A}, P}, were tested without considering migration among the species. The parameter bounds of each population size ranged from 10^{-3} to 100, and those of each divergence time ranged from 0 to 3, as suggested in the ∂a∂i manual (https://dadi.readthedocs.io/en/latest/). The grid size was set to (40, 50, 60). The maximum iteration for an inference was set to 20. Randomly perturbing the initial values using the 'perturb_params' function in ∂a∂i (*32*), the parameters were inferred 100 times. Under these conditions, the {{A, P}, C} model had the highest likelihood out of the three models (Table S4).

Based on the assumption that {{A, P}, C} represents the true evolutionary relationship among the three wild *Dioscorea* species, the evolutionary parameters were re-estimated by ∂a∂i (*32*), allowing symmetric migration among the species. The parameter bounds of each symmetric migration rate ranged from 0 to 20, as also suggested in the ∂a∂i manual. The parameters were inferred 100 times by ∂a∂i (*32*) with different initial parameters, and the best parameter set was selected based on Akaike information criterion.

S4.4 Inferring the evolutionary history of wild *Dioscorea* species using fastsimcoal2

To complement our results and to exactly replicate the conditions used in the previous report (*28*), fastsimcoal2 (*33*), which was used in the previous study (*28*), was also used to test these three models ({{A, P}, C}, {{P, C}, A}, and {{C, A}, P}). Until the SNP calling step, we basically followed our own pipeline in supplementary text S3.2 based on the reference genome version 1 including the unanchored contigs (*1*) to be consistent with the previous study (*28*). The misclassified samples excluding hybrids were genetically re-classified by admixture analysis following the methods used in the previous study (*28*). The threshold of missing rate across all samples was set to 0.25, as proposed in the previous study (*28*). We obtained 87,671 SNPs using our pipeline, fewer than the number of SNPs analyzed in the previous coalescent simulation (*28*). Therefore, we skipped the down-sampling of the SNPs to 100,000, unlike in the previous study (*28*). For the other steps and the parameter bounds for the coalescent simulation by fastsimcoal2 (*33*), we followed the method used in the previous study exactly (*28*) using the same version of fastsimcoal2 (*33*).

S5. Test of hybrid origin

S5.1 Site frequency spectrum polarized by two candidate progenitors of Guinea yam

We focused on the allele frequencies of 388 *D. rotundata* sequences, including 80 from the previous study (*28*), at the SNPs positioned over the entire genome that are oppositely fixed in the two candidate progenitors. The SNP set was generated as described in supplementary text S4.1. Based on this SNP set, 144 SNPs were oppositely fixed in the two candidate progenitors across all pseudo-chromosomes; the allele frequencies of these 144 SNPs were calculated and plotted.

S5.2 Inferring the domestication history of Guinea yam using ∂a∂i

To infer the domestication history of Guinea yam, we used ∂a∂i (*32*). Using the 15,461 polarized SNPs generated by following the method in supplementary text S4.1, three phylogenetic models, {{A, R}, P}, {{P, R}, A}, and {{A, R}, {P, R}} (hypothesis 1, 2, and 3 in Fig. 2A, respectively) were tested, considering symmetric migration among the species. The parameter bound for the admixed proportion from *D. abyssinica* ranged from 0 to 1. The other parameter bounds were the

same as in supplementary text S4.3. The maximum iteration for an inference was set to 20. The parameters were inferred 100 times by ∂a∂i (32).

S5.3 Comparison of F_{ST} on each chromosome among three African yams

F_{ST} (*34*) among the three species (*D. abyssinica*, [Western] *D. praehensilis*, and *D. rotundata*) was calculated in each chromosome. We estimated F_{ST} using the formula:

$$F_{ST} = \frac{H_T - H_S}{H_T}$$

where H_T and H_S are the expected heterozygosity in the total population and sub-divided population, respectively, which are equal to:

$$H_T = 2\frac{f_{A1} + f_{A2}}{2}(1 - \frac{f_{A1} + f_{A2}}{2})$$

$$H_S = \frac{2f_{A1}(1 - f_{A1}) + 2f_{A2}(1 - f_{A2})}{2} = f_{A1}(1 - f_{A1}) + f_{A2}(1 - f_{A2})$$

where f_{A1} and f_{A2} are the allele frequencies in each population (*34*). Finally, the calculated F_{ST} were averaged in each chromosome.

S6. Haplotype network analysis of the whole plastid genome

The sample set used to construct the haplotype network of the whole plastid genome was the same as that used to construct the NJ tree (supplementary text S4.2). We aligned the 458 whole-genome sequences, together with the whole plastid genome of *D. rotundata* (*1*), to the newly improved reference genome of *D. rotundata*. We followed the pipeline described in supplementary text S3.2 for quality control and alignment. Because the plastid genome is haploid, the "--ploidy" option was set to 1 in the BCFtools call command (*17*) when SNPs were called. Singleton SNPs were removed as unreliable markers. SNPs with more than one low-quality genotype (GQ<127) across the samples were also removed as unreliable markers. We did not allow any missing data. Finally, a haplotype network was constructed using the retained 250 SNPs by the median joining network algorithm (*35*) implemented in PopART (*36*).

S7. Inferring the changes in population size

To explore the changes in population sizes, the demographic history of African yams was re-inferred by ∂a∂i (*32*) allowing migration. By fixing the parameters predicted in supplementary text S5.2 except for population sizes, we re-estimated each population size at the start and end points after the emergence of these species, assuming an exponential increase/decrease in population size. The parameter bounds of population sizes ranged from 10^{-3} to 100, and the maximum iteration for an inference was set to 20. The parameters were inferred by ∂a∂i 100 times (*32*).

S8. Exploring the possibility of extensive introgression from *Dioscorea* species

To explore the possibility of multiple introgressions from both parental wild yams, the f_4 statistic (*37*, *38*) was applied to the four clusters of *D. rotundata* excluding the cluster 1 triploid accessions. Here, calculation of the f_4 statistic requires four populations: P_{R1} is the first cluster of *D. rotundata*, P_{R2} is the second cluster of *D. rotundata*, P_P is a population of (Western) *D. praehensilis*, and P_A is a population of *D. abyssinica*. We estimated $\hat{f}_4(P_{R1}, P_{R2}, P_P, P_A)$ with the following formula using sliding window analysis with a window size of 250 kb and a step size of 25 kb:

$$\hat{f}_4(P_{R1}, P_{R2}, P_P, P_A) = (\hat{p}_{R1} - \hat{p}_{R2})(\hat{p}_P - \hat{p}_A)$$

where $\hat{p}_j$ is the observed allele frequency in a window in population P_j.

In most windows, $\hat{f}_4$ is close to zero, which means that the window has a concordant genealogy because the two clusters of *D. rotundata* have a small genetic distance (B in Fig. SM10). However, if these two clusters of *D. rotundata* have a large genetic distance and if one or both populations have a small genetic distance from a wild *Dioscorea* species, then $\hat{f}_4$ skews from 0. Therefore, a locus having a skewed $\hat{f}_4$ has a discordant genealogy (C or D in Fig. SM10). For P_P (the population of *D. praehensilis*) and P_A (the population of *D. abyssinica*), the samples sequenced in the previous study (*28*) were used (Table SM11), and the dataset was prepared as described in supplementary text S4.1. As the first screening, all possible combinations of the clusters of *D. rotundata*, excluding accessions in cluster 1, were used for P_{R1} and P_{R2} (Fig. SM11). In this analysis, we identified an extensive introgression around the *SWEETIE* gene (4.00 to 4.15 Mb on chromosome 17). Because clusters 2 and 5 have the same genealogy pattern around the *SWEETIE* gene, we merged them into one population (P_{25}) and used this as P_{R1}. Because cluster 4 has the opposite genealogy pattern to P_{25} around the *SWEETIE* gene, we used P_4 as P_{R2}. As a result, $\hat{f}_4(P_{25}, P_4, P_P, P_A)$ was calculated for the second screening (Fig. 4). If a locus had $|Z(f_4)|>5$, we regarded it as an outlier (red dots in Fig. 4B). To reveal the relationships of the *D. rotundata* accessions around the *SWEETIE* gene, a Neighbor-Net was constructed by SplitsTree v5.1.4 (*39*) using 308 *D. rotundata* accessions excluding the accessions in cluster 1, 29 *D. abyssinica* accessions, and 21 *D. praehensilis* accessions. A total 458 SNPs from the 4.00–4.15 Mb region on chromosome 17 were converted to multi-FASTA format. At that time, heterozygous genotypes were converted to IUPAC codes.

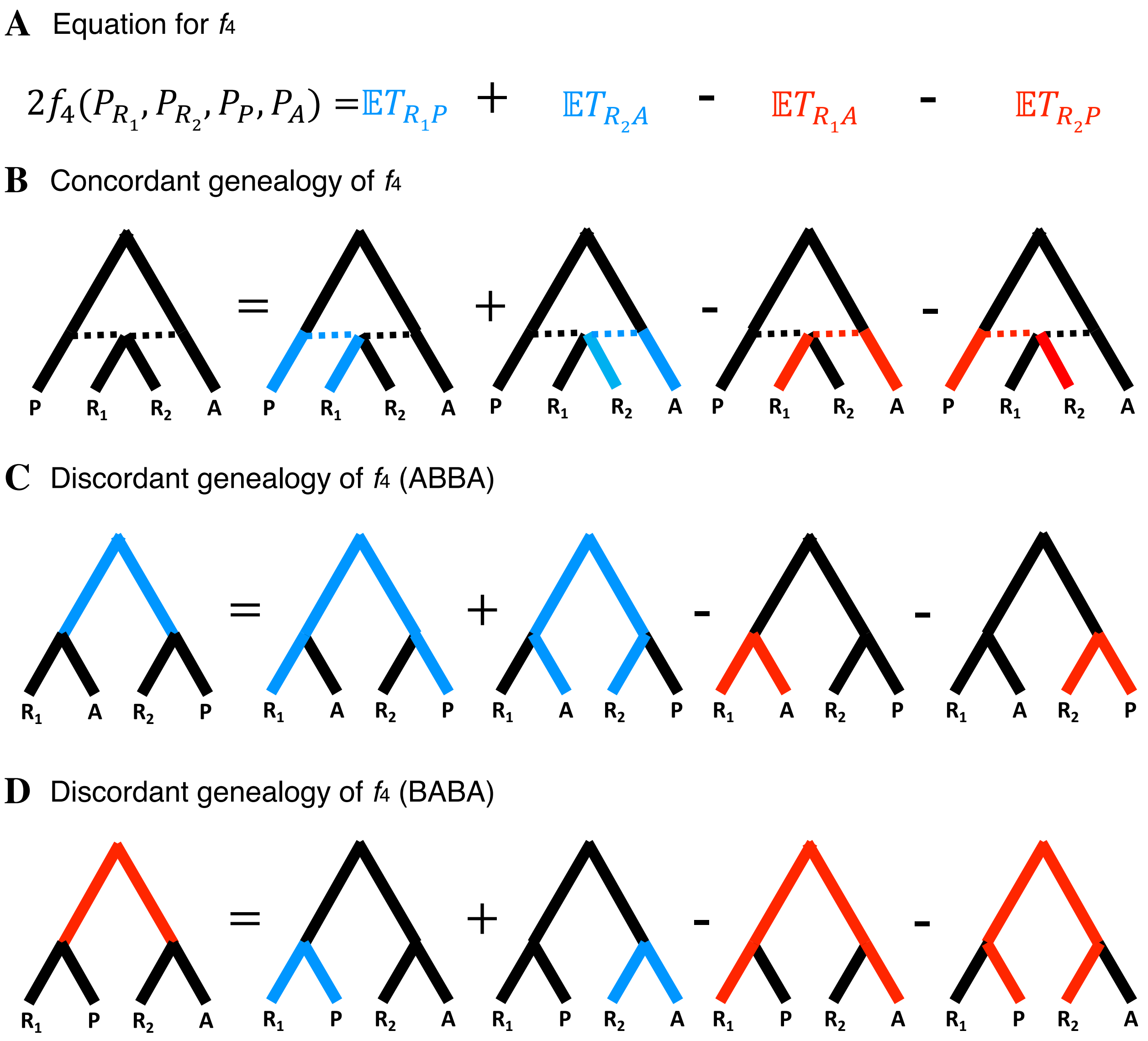

Fig. SM10. Schematic explaining how f_4 behaved in this study. "A" represents the population of *D. abyssinica*. "P" represents the population of *D. praehensilis*. "R1" represents the first populations of *D. rotundata*. "R2" represents the second populations of *D. rotundata*. This figure was adapted from (38).

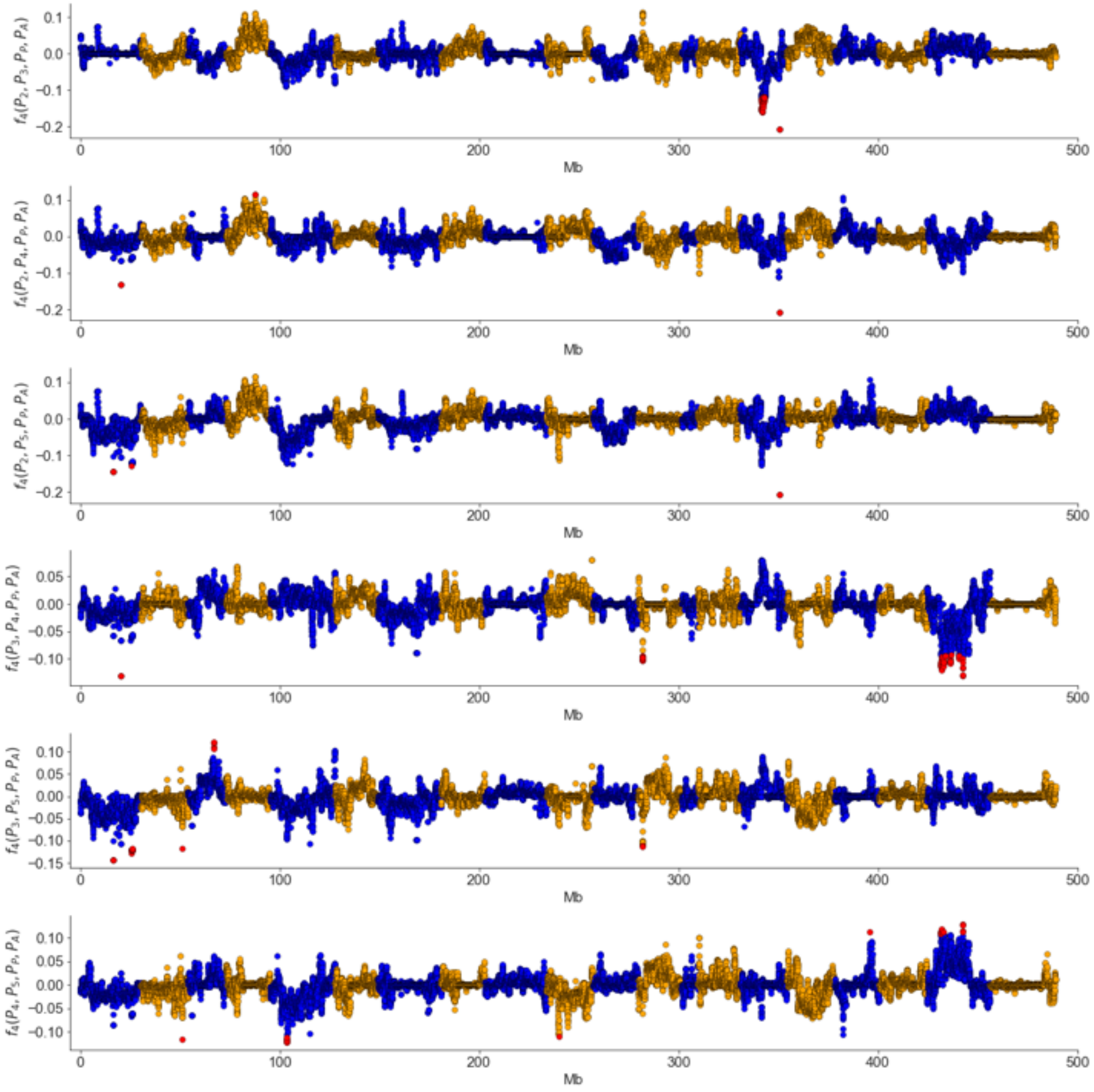

Fig. SM11. f_4 in all possible combinations of clusters excluding cluster 1. Population P*i* represents a population of the cluster *i*.

1. M. Tamiru, S. Natsume, H. Takagi, B. White, H. Yaegashi, M. Shimizu, K. Yoshida, A. Uemura, K. Oikawa, A. Abe, N. Urasaki, H. Matsumura, P. Babil, S. Yamanaka, R. Matsumoto, S. Muranaka, G. Girma, A. Lopez-Montes, M. Gedil, R. Bhattacharjee, M. Abberton, P. L. Kumar, I. Rabbi, M. Tsujimura, T. Terachi, W. Haerty, M. Corpas, S. Kamoun, G. Kahl, H. Takagi, R. Asiedu, R. Terauchi, Genome sequencing of the staple food crop white Guinea yam enables the development of a molecular marker for sex determination. *BMC Biol.* **15**, 86 (2017).
2. W. De Coster, S. D'Hert, D. T. Schultz, M. Cruts, C. Van Broeckhoven, NanoPack: visualizing and processing long-read sequencing data. *Bioinformatics.* **34**, 2666–2669 (2018).
3. M. Kolmogorov, J. Yuan, Y. Lin, P. A. Pevzner, Assembly of long, error-prone reads using repeat graphs. *Nat. Biotechnol.* **37**, 540–546 (2019).

4. F. A. Simão, R. M. Waterhouse, P. Ioannidis, E. V. Kriventseva, E. M. Zdobnov, BUSCO: assessing genome assembly and annotation completeness with single-copy orthologs. *Bioinformatics.* **31**, 3210–3212 (2015).
5. B. J. Walker, T. Abeel, T. Shea, M. Priest, A. Abouelliel, S. Sakthikumar, C. A. Cuomo, Q. Zeng, J. Wortman, S. K. Young, A. M. Earl, Pilon: An Integrated Tool for Comprehensive Microbial Variant Detection and Genome Assembly Improvement. *PLoS ONE.* **9**, e112963 (2014).
6. H. Li, R. Durbin, Fast and accurate short read alignment with Burrows-Wheeler transform. *Bioinformatics*. **25**, 1754–1760 (2009).
7. H. Li, B. Handsaker, A. Wysoker, T. Fennell, J. Ruan, N. Homer, G. Marth, G. Abecasis, R. Durbin, The Sequence Alignment/Map format and SAMtools. *Bioinformatics*. **25**, 2078–2079 (2009).
8. M. J. Roach, S. A. Schmidt, A. R. Borneman, Purge Haplotigs: allelic contig reassignment for third-gen diploid genome assemblies. *BMC Bioinform.* **19**, 460 (2018).
9. C.-C. Lo, P. S. G. Chain, Rapid evaluation and quality control of next generation sequencing data with FaQCs. *BMC Bioinform.* **15**, 366 (2014).
10. D. Kim, B. Langmead, S. L. Salzberg, HISAT: a fast spliced aligner with low memory requirements. *Nat. Methods*. **12**, 357–360 (2015).
11. M. Pertea, G. M. Pertea, C. M. Antonescu, T.-C. Chang, J. T. Mendell, S. L. Salzberg, StringTie enables improved reconstruction of a transcriptome from RNA-seq reads. *Nat. Biotechnol.* **33**, 290–295 (2015).
12. Y. S. Niknafs, B. Pandian, H. K. Iyer, A. M. Chinnaiyan, M. K. Iyer, TACO produces robust multisample transcriptome assemblies from RNA-seq. *Nat. Methods.* **14**, 68–70 (2017).
13. H. Iwata, O. Gotoh, Benchmarking spliced alignment programs including Spaln2, an extended version of Spaln that incorporates additional species-specific features. *Nucleic Acids Res.* **40**, e161 (2012).
14. P. Jones, D. Binns, H.-Y. Chang, M. Fraser, W. Li, C. McAnulla, H. McWilliam, J. Maslen, A. Mitchell, G. Nuka, S. Pesseat, A. F. Quinn, A. Sangrador-Vegas, M. Scheremetjew, S.-Y. Yong, R. Lopez, S. Hunter, InterProScan 5: genome-scale protein function classification. *Bioinformatics*. **30**, 1236–1240 (2014).
15. C. Camacho, G. Coulouris, V. Avagyan, N. Ma, J. Papadopoulos, K. Bealer, T. L. Madden, BLAST+: architecture and applications. *BMC Bioinformatics*. **10**, 421 (2009).
16. R. Schmieder, R. Edwards, Quality control and preprocessing of metagenomic datasets. *Bioinformatics*. **27**, 863–864 (2011).
17. H. Li, A statistical framework for SNP calling, mutation discovery, association mapping and population genetical parameter estimation from sequencing data. *Bioinformatics*. **27**, 2987–2993 (2011).
18. A. R. Quinlan, I. M. Hall, BEDTools: a flexible suite of utilities for comparing genomic features. *Bioinformatics.* **26**, 841–842 (2010).
19. Y. Wu, P. R. Bhat, T. J. Close, S. Lonardi, Efficient and Accurate Construction of Genetic Linkage Maps from the Minimum Spanning Tree of a Graph. *PLoS Genet.* **4**, e1000212 (2008).
20. K. W. Broman, H. Wu, S. Sen, G. A. Churchill, R/qtl: QTL mapping in experimental crosses. *Bioinformatics*. **19**, 889–890 (2003).
21. F. Cabanettes, C. Klopp, D-GENIES: dot plot large genomes in an interactive, efficient and simple way. *PeerJ*. **6**, e4958 (2018).
22. X. Zheng, D. Levine, J. Shen, S. M. Gogarten, C. Laurie, B. S. Weir, A high-performance computing toolset for relatedness and principal component analysis of SNP data. *Bioinformatics*. **28**, 3326–3328 (2012).
23. E. Frichot, F. Mathieu, T. Trouillon, G. Bouchard, O. François, Fast and Efficient Estimation of Individual Ancestry Coefficients. *Genetics*. **196**, 973–983 (2014).

24. S. R. Browning, B. L. Browning, Rapid and Accurate Haplotype Phasing and Missing-Data Inference for Whole-Genome Association Studies By Use of Localized Haplotype Clustering. *Am. J. Hum. Genet.* **81**, 1084–1097 (2007).
25. G. A. Watterson, On the number of segregating sites in genetical models without recombination. *Theor. Popul. Biol.* **7**, 256–276 (1975).
26. M. Nei, F. Tajima, DNA polymorphism detectable by restriction endonucleases. *Genetics*. **97**, 145–167 (1981).
27. F. Tajima, Statistical method for testing the neutral mutation hypothesis by DNA polymorphism. *Genetics*. **123**, 585–595 (1989).
28. N. Scarcelli, P. Cubry, R. Akakpo, A. C. Thuillet, J. Obidiegwu, M. N. Baco, E. Otoo, B. Sonké, A. Dansi, G. Djedatin, C. Mariac, M. Couderc, S. Causse, K. Alix, H. Chaïr, O. François, Y. Vigouroux, Yam genomics supports West Africa as a major cradle of crop domestication. *Sci. Adv.* **5**, eaaw1947 (2019).
29. N. Saitou, M Nei, The neighbor-joining method: a new method for reconstructing phylogenetic trees. *Mol. Biol. Evol.* **4**, 406–425 (1987).
30. S. Kumar, G. Stecher, M. Li, C. Knyaz, K. Tamura, MEGA X: Molecular Evolutionary Genetics Analysis across Computing Platforms. *Mol. Biol. Evol.* **35**, 1547–1549 (2018).
31. G. Yu, D. K. Smith, H. Zhu, Y. Guan, T. T. Lam, GGTREE: an R package for visualization and annotation of phylogenetic trees with their covariates and other associated data. *Methods Ecol. Evol.* **8**, 28–36 (2017).
32. R. N. Gutenkunst, R. D. Hernandez, S. H. Williamson, C. D. Bustamante, Inferring the Joint Demographic History of Multiple Populations from Multidimensional SNP Frequency Data. *PLoS Genet*. **5**, e1000695 (2009).
33. L. Excoffier, I. Dupanloup, E. Huerta-Sánchez, V. C. Sousa, M. Foll, Robust demographic Inference from genomic and SNP data. *PLoS Genet*. **9**, e1003905 (2013).
34. S. Wright, The genetical structure of populations. *Ann. Eugen.* **15**, 323–354 (1951).
35. H. J. Bandelt, P. Forster, A. Röhl, Median-joining networks for inferring intraspecific phylogenies. *Mol. Biol. Evol*. **16**, 37–48 (1999).
36. J. W. Leigh, D. Bryant, popart: full-feature software for haplotype network construction. *Methods Ecol. Evol.* **6**, 1110–1116 (2015).
37. D. Reich, K. Thangaraj, N. Patterson, A. L. Price, L. Singh, Reconstructing Indian population history. *Nature.* **461**, 489–494 (2009).
38. B. M. Peter, Admixture, Population Structure, and F-Statistics. *Genetics.* **202**, 1485–1501 (2016).
39. D. H. Huson, D. Bryant, Application of Phylogenetic Networks in Evolutionary Studies. *Mol. Biol. Evol.* **23**, 254–267 (2006).
40. N. Scarcelli, H. Chaïr, S. Causse, R. Vesta, T. L. Couvreur, Y. Vigouroux, Crop wild relative conservation: Wild yams are not that wild. *Biol. Conserv*. **210**, 325–333 (2017).

Table SM7. Summary of sequence alignment of mapping population

Sample		Fastq size		Aligned bam information						
Name	IITA name	Original (Gbp)	Filtered (Gbp)	Aligned (Gbp)	Unmapped (Gbp)	Coverage (%)	Depth	Sequence platform	Comment	Accession No.
TDr04_219	TDr04_219	38.26	33.10	17.15	0.32	82.8	35.73	MiSeq, HiSeq4000, GAIIx	MP2 family Mono parent	DRR208404,DRR208405,DRR063085
TDr97_777	TDr97_777	25.47	22.71	11.20	0.29	79.4	24.35	MiSeq,HiSeq4000,NextSeq500,GAIIx	MP2 family Male parent	DRR063127,DRR208406,DRR045130-7,DRR063111
MP2_001	MP2_001	8.20	7.14	4.20	1.00	76.9	9.43	HiSeq4000	-	DRR208407
MP2_002	MP2_002	6.42	5.61	3.45	0.64	73.2	8.13	HiSeq4000	-	DRR208408
MP2_003	MP2_003	5.95	5.11	2.92	0.87	71.6	7.03	HiSeq4000	-	DRR208409
MP2_004	MP2_004	7.13	6.24	3.90	0.70	74.8	8.99	HiSeq4000	-	DRR208410
MP2_005	MP2_005	9.75	8.49	4.59	1.56	75.2	10.53	HiSeq4000	-	DRR208411
MP2_006	MP2_006	7.90	7.01	4.39	0.76	77.2	9.80	HiSeq4000	-	DRR208412
MP2_007	MP2_007	7.50	6.57	4.11	0.75	75.8	9.35	HiSeq4000	-	DRR208413
MP2_008	MP2_008	7.52	6.60	3.93	0.81	74.3	9.13	HiSeq4000	-	DRR208414
MP2_009	MP2_009	7.36	6.48	4.12	0.62	76.3	9.33	HiSeq4000	-	DRR208415
MP2_010	MP2_010	6.49	5.72	3.66	0.55	75.2	8.39	HiSeq4000	-	DRR208416
MP2_011	MP2_011	5.98	5.28	3.41	0.49	77.1	7.63	HiSeq4000	-	DRR208417
MP2_012	MP2_012	8.25	7.31	4.69	0.77	76.9	10.53	HiSeq4000	-	DRR208418
MP2_013	MP2_013	9.33	8.05	4.81	1.00	76.2	10.89	HiSeq4000	-	DRR208419
MP2_014	MP2_014	9.84	8.65	5.56	0.81	78.0	12.32	HiSeq4000	-	DRR208420
MP2_015	MP2_015	11.21	9.80	6.29	0.93	78.5	13.82	HiSeq4000	-	DRR208421
MP2_016	MP2_016	12.97	11.36	6.86	1.18	78.1	15.15	HiSeq4000	-	DRR208422
MP2_017	MP2_017	3.89	2.96	1.48	0.36	67.0	3.83	HiSeq4000	-	DRR208423
MP2_018	MP2_018	12.70	11.17	7.04	1.10	78.3	15.53	HiSeq4000	-	DRR208424
MP2_019	MP2_019	5.00	4.31	2.32	0.41	74.2	5.38	HiSeq4000	-	DRR208425
MP2_020	MP2_020	10.13	9.04	6.04	0.78	78.1	13.34	HiSeq4000	-	DRR208426
MP2_023	MP2_023	4.98	3.90	2.10	0.35	71.4	5.08	HiSeq4000	-	DRR208427
MP2_024	MP2_024	10.08	8.74	5.10	1.27	75.4	11.68	HiSeq4000	-	DRR208428
MP2_025	MP2_025	4.80	3.53	1.91	0.38	70.2	4.70	HiSeq4000	-	DRR208429
MP2_026	MP2_026	8.36	7.38	4.88	0.66	77.5	10.86	HiSeq4000	-	DRR208430
MP2_027	MP2_027	5.35	3.86	2.05	0.37	71.6	4.93	HiSeq4000	-	DRR208431
MP2_028	MP2_028	8.11	7.08	4.45	0.72	76.4	10.05	HiSeq4000	-	DRR208432
MP2_029	MP2_029	9.89	8.61	5.03	1.08	75.4	11.52	HiSeq4000	-	DRR208433
MP2_031	MP2_031	10.33	9.08	6.04	0.79	78.5	13.30	HiSeq4000	-	DRR208434
MP2_032	MP2_032	16.56	12.57	6.45	1.21	78.9	14.12	HiSeq4000	-	DRR208435
MP2_033	MP2_033	7.32	6.41	4.19	0.62	77.5	9.34	HiSeq4000	-	DRR208436
MP2_034	MP2_034	8.05	6.99	4.40	0.79	75.0	10.12	HiSeq4000	-	DRR208437
MP2_035	MP2_035	9.06	7.95	4.96	0.83	77.3	11.07	HiSeq4000	-	DRR208438
MP2_037	MP2_037	9.70	8.41	5.16	0.99	77.3	11.53	HiSeq4000	-	DRR208439
MP2_039	MP2_039	7.54	6.58	4.00	0.82	75.4	9.17	HiSeq4000	-	DRR208440
MP2_043	MP2_043	9.15	7.93	4.24	0.71	77.3	9.46	HiSeq4000	-	DRR208441
MP2_044	MP2_044	9.75	8.60	5.28	0.95	76.9	11.85	HiSeq4000	-	DRR208442
MP2_047	MP2_047	8.95	7.64	4.04	0.76	77.1	9.03	HiSeq4000	-	DRR208443
MP2_048	MP2_048	8.27	7.24	3.94	0.69	77.4	8.80	HiSeq4000	-	DRR208444
MP2_050	MP2_050	11.17	9.77	5.67	1.35	76.2	12.85	HiSeq4000	-	DRR208445
MP2_052	MP2_052	9.98	8.75	5.18	1.13	75.1	11.90	HiSeq4000	-	DRR208446
MP2_053	MP2_053	11.85	9.88	4.74	2.21	72.0	11.37	HiSeq4000	-	DRR208447
MP2_054	MP2_054	10.38	6.95	3.67	0.70	77.1	8.21	HiSeq4000	-	DRR208448
MP2_055	MP2_055	12.74	10.66	5.55	1.85	74.8	12.81	HiSeq4000	-	DRR208449

MP2_057	MP2_057	8.68	7.41	4.06	1.24	72.2	9.72	HiSeq4000	-	DRR208450
MP2_058	MP2_058	11.14	9.54	6.10	0.89	78.2	13.47	HiSeq4000	-	DRR208451
MP2_060	MP2_060	8.31	7.05	3.51	0.79	76.0	7.97	HiSeq4000	-	DRR208452
MP2_061	MP2_061	12.07	10.38	6.88	0.95	79.0	15.04	HiSeq4000	-	DRR208453
MP2_063	MP2_063	7.03	5.43	2.96	0.51	76.3	6.71	HiSeq4000	-	DRR208454
MP2_064	MP2_064	11.23	9.50	5.46	1.28	76.0	12.39	HiSeq4000	-	DRR208455
MP2_113	MP2_113	6.79	5.71	3.29	0.79	75.0	7.57	HiSeq4000	-	DRR208456
MP2_114	MP2_114	7.80	6.62	3.60	0.94	70.9	8.75	HiSeq4000	-	DRR208457
MP2_116	MP2_116	7.17	6.14	3.78	0.66	75.5	8.64	HiSeq4000	-	DRR208458
MP2_117	MP2_117	6.52	5.53	3.38	0.55	75.9	7.69	HiSeq4000	-	DRR208459
MP2_121	MP2_121	11.64	10.04	5.72	1.45	76.1	12.96	HiSeq4000	-	DRR208460
MP2_122	MP2_122	9.07	7.65	4.33	1.15	75.5	9.89	HiSeq4000	-	DRR208461
MP2_125	MP2_125	9.25	8.04	4.87	0.86	77.7	10.82	HiSeq4000	-	DRR208462
MP2_126	MP2_126	8.65	7.46	4.36	1.00	76.1	9.89	HiSeq4000	-	DRR208463
MP2_127	MP2_127	11.45	9.94	6.22	0.99	78.0	13.76	HiSeq4000	-	DRR208464
MP2_128	MP2_128	10.17	8.91	5.41	1.01	77.1	12.11	HiSeq4000	-	DRR208465
MP2_129	MP2_129	11.75	10.05	5.97	1.32	77.4	13.30	HiSeq4000	-	DRR208466
MP2_130	MP2_130	9.04	7.78	4.94	0.75	76.8	11.10	HiSeq4000	-	DRR208467
MP2_131	MP2_131	10.02	8.69	5.59	0.85	78.2	12.34	HiSeq4000	-	DRR208468
MP2_132	MP2_132	9.93	8.56	5.23	0.99	77.2	11.69	HiSeq4000	-	DRR208469
MP2_133	MP2_133	7.97	6.87	4.29	0.71	77.0	9.63	HiSeq4000	-	DRR208470
MP2_136	MP2_136	9.56	8.20	4.48	1.48	76.2	10.14	HiSeq4000	-	DRR208471
MP2_137	MP2_137	10.99	9.51	5.70	1.15	76.5	12.86	HiSeq4000	-	DRR208472
MP2_138	MP2_138	8.51	7.42	4.61	0.76	77.3	10.28	HiSeq4000	-	DRR208473
MP2_139	MP2_139	9.41	8.27	5.12	0.83	75.9	11.65	HiSeq4000	-	DRR208474
MP2_140	MP2_140	8.91	7.74	4.74	0.90	76.9	10.65	HiSeq4000	-	DRR208475
MP2_141	MP2_141	9.22	7.61	4.05	1.22	72.2	9.69	HiSeq4000	-	DRR208476
MP2_142	MP2_142	10.72	9.12	4.11	2.49	73.3	9.67	HiSeq4000	-	DRR208477
MP2_143	MP2_143	7.99	6.94	4.03	0.91	75.3	9.24	HiSeq4000	-	DRR208478
MP2_144	MP2_144	9.30	8.14	5.31	0.79	77.5	11.83	HiSeq4000	-	DRR208479
MP2_145	MP2_145	10.35	8.99	5.13	1.17	76.5	11.56	HiSeq4000	-	DRR208480
MP2_146	MP2_146	10.87	9.44	5.39	1.41	77.1	12.07	HiSeq4000	-	DRR208481
MP2_147	MP2_147	9.96	8.80	5.79	0.76	78.4	12.75	HiSeq4000	-	DRR208482
MP2_149	MP2_149	9.80	8.64	5.74	0.78	78.0	12.71	HiSeq4000	-	DRR208483
MP2_150	MP2_150	7.47	6.31	3.17	1.22	71.5	7.65	HiSeq4000	-	DRR208484
MP2_151	MP2_151	8.96	7.85	4.80	0.90	78.0	10.63	HiSeq4000	-	DRR208485
MP2_152	MP2_152	12.30	10.66	6.41	1.29	78.8	14.02	HiSeq4000	-	DRR208486
MP2_154	MP2_154	9.78	8.41	4.56	1.42	75.8	10.38	HiSeq4000	-	DRR208487
MP2_155	MP2_155	10.40	9.01	5.31	1.23	77.5	11.82	HiSeq4000	-	DRR208488
MP2_156	MP2_156	8.67	7.49	4.32	1.00	76.2	9.79	HiSeq4000	-	DRR208489
MP2_157	MP2_157	7.64	6.64	4.00	0.84	76.0	9.08	HiSeq4000	-	DRR208490
MP2_158	MP2_158	8.84	7.67	4.85	0.79	77.8	10.77	HiSeq4000	-	DRR208491
MP2_159	MP2_159	9.82	8.47	4.97	1.16	77.2	11.10	HiSeq4000	-	DRR208492
MP2_160	MP2_160	8.43	7.33	4.57	0.73	77.2	10.23	HiSeq4000	-	DRR208493
MP2_161	MP2_161	8.93	7.71	4.46	1.10	77.1	9.99	HiSeq4000	-	DRR208494
MP2_162	MP2_162	12.11	10.46	5.71	1.62	77.4	12.73	HiSeq4000	-	DRR208495
MP2_166	MP2_166	12.03	10.49	6.27	1.21	76.7	14.09	HiSeq4000	-	DRR208496
MP2_167	MP2_167	9.67	8.39	4.63	1.31	74.7	10.70	HiSeq4000	-	DRR208497
MP2_168	MP2_168	15.43	13.47	8.68	1.28	79.0	18.96	HiSeq4000	-	DRR208498

MP2_169	MP2_169	12.87	11.15	6.58	1.40	77.7	14.62	HiSeq4000	-	DRR208499
MP2_170	MP2_170	13.20	11.31	6.24	1.83	77.3	13.94	HiSeq4000	-	DRR208500
MP2_172	MP2_172	11.50	9.60	5.68	1.08	75.6	12.97	HiSeq4000	-	DRR208501
MP2_173	MP2_173	10.20	8.86	4.90	1.31	74.9	11.28	HiSeq4000	-	DRR208502
MP2_174	MP2_174	10.70	9.28	5.37	1.26	77.7	11.95	HiSeq4000	-	DRR208503
MP2_175	MP2_175	13.09	11.51	7.00	1.21	77.4	15.60	HiSeq4000	-	DRR208504
MP2_177	MP2_177	6.33	5.38	2.88	1.00	71.7	6.93	HiSeq4000	-	DRR208505
MP2_178	MP2_178	5.89	5.10	3.00	0.66	73.2	7.07	HiSeq4000	-	DRR208506
MP2_179	MP2_179	4.55	3.89	2.47	0.42	73.5	5.79	HiSeq4000	-	DRR208507
MP2_180	MP2_180	7.09	6.10	3.54	0.86	74.8	8.17	HiSeq4000	-	DRR208508
MP2_181	MP2_181	6.41	5.45	3.05	0.91	72.6	7.26	HiSeq4000	-	DRR208509
MP2_182	MP2_182	8.34	7.16	4.72	0.71	78.2	10.42	HiSeq4000	-	DRR208510
MP2_183	MP2_183	8.89	7.74	5.12	0.74	77.0	11.47	HiSeq4000	-	DRR208511
MP2_185	MP2_185	6.46	5.49	3.06	0.97	72.4	7.30	HiSeq4000	-	DRR208512
MP2_186	MP2_186	6.37	5.37	3.39	0.59	76.0	7.70	HiSeq4000	-	DRR208513
MP2_187	MP2_187	5.86	4.97	2.86	0.72	72.4	6.83	HiSeq4000	-	DRR208514
MP2_188	MP2_188	8.36	7.11	4.48	0.83	76.4	10.12	HiSeq4000	-	DRR208515
MP2_189	MP2_189	6.63	5.69	3.34	0.75	73.9	7.80	HiSeq4000	-	DRR208516
MP2_190	MP2_190	6.41	5.35	3.44	0.58	77.4	7.67	HiSeq4000	-	DRR208517
MP2_191	MP2_191	7.46	6.22	3.76	0.85	74.9	8.67	HiSeq4000	-	DRR208518
MP2_192	MP2_192	6.76	5.71	3.54	0.64	74.8	8.16	HiSeq4000	-	DRR208519
MP2_193	MP2_193	9.63	8.56	5.41	0.86	77.5	12.06	HiSeq4000	-	DRR208520
MP2_196	MP2_196	11.11	9.85	6.23	0.96	78.2	13.76	HiSeq4000	-	DRR208521
MP2_197	MP2_197	7.35	6.22	3.96	0.66	76.6	8.92	HiSeq4000	-	DRR208522
MP2_198	MP2_198	8.72	7.48	4.86	0.74	78.2	10.74	HiSeq4000	-	DRR208523
MP2_199	MP2_199	6.66	5.90	3.58	0.69	74.8	8.25	HiSeq4000	-	DRR208524
MP2_200	MP2_200	7.00	6.22	3.99	0.61	75.8	9.08	HiSeq4000	-	DRR208525
MP2_201	MP2_201	8.36	7.17	4.39	0.86	75.4	10.06	HiSeq4000	-	DRR208526
MP2_202	MP2_202	9.03	7.71	3.83	1.87	74.4	8.88	HiSeq4000	-	DRR208527
MP2_203	MP2_203	7.58	6.73	4.06	0.76	76.8	9.12	HiSeq4000	-	DRR208528
MP2_204	MP2_204	10.55	9.21	5.02	1.48	77.2	11.22	HiSeq4000	-	DRR208529
MP2_205	MP2_205	11.71	10.10	6.18	1.22	77.5	13.76	HiSeq4000	-	DRR208530
MP2_206	MP2_206	8.72	7.29	3.94	1.43	74.1	9.16	HiSeq4000	-	DRR208531
MP2_208	MP2_208	11.54	10.28	6.41	1.12	78.2	14.16	HiSeq4000	-	DRR208532
MP2_211	MP2_211	9.81	8.70	5.44	1.02	78.4	11.98	HiSeq4000	-	DRR208533
MP2_213	MP2_213	10.05	8.77	5.30	1.02	78.0	11.73	HiSeq4000	-	DRR208534
MP2_214	MP2_214	8.64	7.69	4.64	0.96	76.1	10.53	HiSeq4000	-	DRR208535
MP2_215	MP2_215	9.92	8.76	5.62	0.81	78.0	12.43	HiSeq4000	-	DRR208536
MP2_216	MP2_216	9.92	8.64	5.19	1.10	75.4	11.88	HiSeq4000	-	DRR208537
MP2_218	MP2_218	9.62	8.52	5.24	1.10	75.4	11.99	HiSeq4000	-	DRR208538
MP2_219	MP2_219	7.57	6.57	4.15	0.70	74.8	9.57	HiSeq4000	-	DRR208539
MP2_220	MP2_220	7.81	6.90	4.21	0.78	76.1	9.55	HiSeq4000	-	DRR208540
MP2_221	MP2_221	9.33	8.28	5.13	0.92	76.2	11.63	HiSeq4000	-	DRR208541
MP2_222	MP2_222	9.13	7.90	4.79	1.02	75.7	10.93	HiSeq4000	-	DRR208542
MP2_224	MP2_224	11.19	9.85	6.23	1.05	77.1	13.95	HiSeq4000	-	DRR208543
MP2_225	MP2_225	8.97	7.74	4.41	1.09	74.2	10.25	HiSeq4000	-	DRR208544
MP2_227	MP2_227	14.19	12.43	7.97	1.15	78.7	17.48	HiSeq4000	-	DRR208545
MP2_228	MP2_228	9.03	7.86	4.92	0.90	76.8	11.05	HiSeq4000	-	DRR208546
MP2_229	MP2_229	10.39	9.13	5.71	0.97	77.5	12.73	HiSeq4000	-	DRR208547

MP2_231	MP2_231	10.31	8.99	5.62	0.96	77.6	12.50	HiSeq4000	-	DRR208548
MP2_232	MP2_232	11.06	9.64	6.00	1.04	77.1	13.41	HiSeq4000	-	DRR208549
MP2_233	MP2_233	9.57	8.46	5.23	1.07	76.8	11.76	HiSeq4000	-	DRR208550
MP2_234	MP2_234	6.96	6.02	3.42	0.89	73.4	8.05	HiSeq4000	-	DRR208551
MP2_235	MP2_235	8.71	7.54	4.21	1.25	73.9	9.82	HiSeq4000	-	DRR208552
MP2_236	MP2_236	5.82	4.95	3.06	0.56	73.8	7.16	HiSeq4000	-	DRR208553
MP2_237	MP2_237	6.46	5.55	3.27	0.80	74.2	7.61	HiSeq4000	-	DRR208554
MP2_239	MP2_239	7.08	6.14	3.77	0.73	75.0	8.66	HiSeq4000	-	DRR208555
MP2_240	MP2_240	6.92	6.00	3.70	0.78	74.4	8.59	HiSeq4000	-	DRR208556
MP2_241	MP2_241	10.28	8.87	4.73	1.60	74.7	10.92	HiSeq4000	-	DRR208557
MP2_242	MP2_242	8.82	7.65	4.62	0.85	75.3	10.58	HiSeq4000	-	DRR208558
MP2_245	MP2_245	5.90	5.15	3.32	0.51	76.3	7.50	HiSeq4000	-	DRR208559
MP2_246	MP2_246	6.86	5.98	3.77	0.70	76.6	8.50	HiSeq4000	-	DRR208560
MP2_247	MP2_247	6.97	6.01	3.70	0.65	74.3	8.61	HiSeq4000	-	DRR208561
MP2_248	MP2_248	6.45	5.60	3.62	0.57	76.7	8.14	HiSeq4000	-	DRR208562

Table SM11. All sequence information of ourgroups.

Sample		Fastq size		Aligned bam information					
Name	Name in Scarcelli et al. 2019	Original (Gbp)	Filtered (Gbp)	Aligned (Gbp)	Unmapped (Gbp)	Coverage (%)	Depth	Comment	Accession No.
alata1		28.11	23.95	10.73	1.24	48.0	38.59	D.alata	ERR1019033
alata2		11.58	11.15	3.88	1.37	43.1	15.54	D.alata	SRR7062294
ns004_A5689	A5689	4.22	4.19	3.09	0.34	75.2	7.09	D.abyssinica:Nigeria	SRR8451439
ns005_A5690	A5690	5.79	5.72	4.06	0.37	68.5	10.24	D.abyssinica:Nigeria	SRR8451438
ns006_A5691	A5691	5.53	5.49	2.85	1.73	68.4	7.20	D.abyssinica:Nigeria	SRR8451437
ns007_A5693	A5693	5.93	5.89	4.54	0.15	78.3	10.01	D.abyssinica:Nigeria	SRR8451434
ns008_A5694	A5694	4.87	4.84	3.91	0.04	77.3	8.72	D.abyssinica:Nigeria	SRR8451371
ns009_A5695	A5695	4.55	4.52	3.35	0.42	78.4	7.37	D.abyssinica:Nigeria	SRR8451459
ns010_A5696	A5696	4.75	4.61	3.55	0.22	74.9	8.17	D.abyssinica:Nigeria	SRR8451458
ns011_A5697	A5697	5.70	5.66	4.41	0.15	80.2	9.48	D.abyssinica:Nigeria	SRR8451382
ns012_A5699	A5699	3.25	3.22	2.45	0.15	71.8	5.89	D.abyssinica:Nigeria	SRR8451381
ns013_A5700	A5700	4.79	4.76	3.59	0.32	77.0	8.05	D.abyssinica:Nigeria	SRR8451384
ns014_A5701	A5701	5.99	5.95	4.38	0.37	78.6	9.62	D.abyssinica:Nigeria	SRR8451383
ns015_A5702	A5702	3.96	3.93	2.95	0.29	74.9	6.79	D.abyssinica:Nigeria	SRR8451378
ns016_A5703	A5703	4.53	4.49	3.09	0.37	65.3	8.17	D.abyssinica:Nigeria	SRR8451377
ns017_A5704	A5704	4.95	4.91	2.85	1.17	69.6	7.08	D.abyssinica:Nigeria	SRR8451380
ns018_A5705	A5705	5.54	5.49	3.75	0.67	74.5	8.68	D.abyssinica:Nigeria	SRR8451379
ns019_A52	A52	1.66	1.63	1.44	0.02	70.8	3.52	D.abyssinica:Benin	SRR8451376
ns020_A62	A62	2.35	2.31	2.06	0.02	77.3	4.60	D.abyssinica:Benin	SRR8451375
ns021_A67	A67	7.54	7.42	6.12	0.12	85.2	12.40	D.abyssinica:Benin	SRR8451343
ns023_A467	A467	5.72	5.64	5.08	0.06	82.0	10.69	D.abyssinica:Benin	SRR8451345
ns024_A537	A537	6.22	6.13	5.28	0.05	79.3	11.49	D.abyssinica:Benin	SRR8451346
ns025_A3009	A3009	3.33	3.27	2.92	0.03	76.7	6.57	D.abyssinica:Benin	SRR8451347
ns027_A5068	A5068	1.98	1.95	1.67	0.04	65.7	4.38	D.abyssinica:Ghana	SRR8451349
ns028_A5045	A5045	2.61	2.56	2.21	0.04	74.4	5.12	D.abyssinica:Ghana	SRR8451350
ns029_A5047	A5047	3.32	3.27	2.80	0.04	75.0	6.46	D.abyssinica:Ghana	SRR8451351
ns030_A5048	A5048	9.39	9.23	7.75	0.10	82.9	16.14	D.abyssinica:Ghana	SRR8451352
ns031_A5059	A5059	10.28	10.10	7.09	1.66	82.5	14.82	D.abyssinica:Ghana	SRR8451320
ns032_A5061	A5061	2.81	2.77	1.91	0.54	72.4	4.55	D.abyssinica:Ghana	SRR8451319
ns033_A5066	A5066	8.09	7.95	6.74	0.11	80.7	14.41	D.abyssinica:Ghana	SRR8451318
ns034_A5067	A5067	7.67	7.55	6.51	0.06	82.0	13.71	D.abyssinica:Ghana	SRR8451317
ns035_P5344	P5344	3.33	3.30	2.46	0.10	70.6	6.02	D.praehensilis:Cameroon:Cameroonian D.praehensilis	SRR8451316
ns036_P5350	P5350	4.06	4.02	2.77	0.20	63.5	7.52	D.praehensilis:Cameroon:Cameroonian D.praehensilis	SRR8451315
ns037_P5358	P5358	4.21	4.17	3.09	0.15	73.2	7.29	D.praehensilis:Cameroon:Cameroonian D.praehensilis	SRR8451314
ns038_P5369	P5369	3.10	3.08	2.17	0.32	70.2	5.34	D.praehensilis:Cameroon:Cameroonian D.praehensilis	SRR8451313
ns039_P5378	P5378	3.01	2.99	2.31	0.05	70.5	5.66	D.praehensilis:Cameroon:Cameroonian D.praehensilis	SRR8451322
ns040_P5381	P5381	3.90	3.87	2.97	0.11	72.8	7.05	D.praehensilis:Cameroon:Cameroonian D.praehensilis	SRR8451321

ns041_P5404	P5404	4.53	4.49	3.31	0.31	74.3	7.69	D.praehensilis:Cameroon:Cameroonian D.praehensilis	SRR8451462
ns042_P5413	P5413	3.78	3.75	2.82	0.16	73.5	6.62	D.praehensilis:Cameroon:Cameroonian D.praehensilis	SRR8451463
ns043_P5417	P5417	4.61	4.58	3.44	0.19	74.1	8.01	D.praehensilis:Cameroon:Cameroonian D.praehensilis	SRR8451460
ns044_P5420	P5420	2.25	2.23	1.65	0.15	65.9	4.31	D.praehensilis:Cameroon:Cameroonian D.praehensilis	SRR8451461
ns045_P5424	P5424	5.30	5.26	3.74	0.42	74.4	8.68	D.praehensilis:Cameroon:Cameroonian D.praehensilis	SRR8451466
ns046_P5427	P5427	4.25	4.22	3.24	0.05	72.9	7.66	D.praehensilis:Cameroon:Cameroonian D.praehensilis	SRR8451467
ns047_P5430	P5430	3.34	3.31	2.41	0.10	63.5	6.56	D.praehensilis:Cameroon:Cameroonian D.praehensilis	SRR8451464
ns048_P5434	P5434	2.80	2.77	2.10	0.06	61.8	5.86	D.praehensilis:Cameroon:Cameroonian D.praehensilis	SRR8451465
ns049_P5438	P5438	3.64	3.61	2.36	0.62	70.6	5.76	D.praehensilis:Cameroon:Cameroonian D.praehensilis	SRR8451468
ns050_P5441	P5441	4.13	4.09	3.04	0.23	73.7	7.12	D.praehensilis:Cameroon:Cameroonian D.praehensilis	SRR8451469
ns051_P5448	P5448	4.73	4.69	3.66	0.09	73.6	8.58	D.praehensilis:Cameroon:Cameroonian D.praehensilis	SRR8451449
ns054_P5318	P5318	5.04	4.99	3.07	0.62	67.7	7.83	D.praehensilis:Cameroon:Cameroonian D.praehensilis	SRR8451450
ns055_P5746	P5746	3.80	3.77	2.66	0.43	65.3	7.02	D.praehensilis:Nigeria:Western D.praehensilis	SRR8451453
ns056_P5708	P5708	6.19	6.13	4.22	0.39	64.5	11.30	D.praehensilis:Nigeria:Western D.praehensilis	SRR8451452
ns057_P5710	P5710	3.89	3.86	2.61	0.48	70.0	6.42	D.praehensilis:Nigeria:Western D.praehensilis	SRR8451455
ns058_P5713	P5713	3.24	3.21	2.34	0.22	67.2	6.02	D.praehensilis:Nigeria:Western D.praehensilis	SRR8451454
ns059_P5716	P5716	2.56	2.53	1.91	0.03	63.0	5.23	D.praehensilis:Nigeria:Western D.praehensilis	SRR8451457
ns061_P5720	P5720	3.87	3.84	2.99	0.17	73.5	7.02	D.praehensilis:Nigeria:Western D.praehensilis	SRR8451430
ns062_P5723	P5723	3.63	3.61	2.17	0.93	68.9	5.44	D.praehensilis:Nigeria:Western D.praehensilis	SRR8451431
ns063_P5728	P5728	3.75	3.71	2.65	0.34	64.3	7.11	D.praehensilis:Nigeria:Western D.praehensilis	SRR8451432
ns064_P5729	P5729	7.31	7.25	4.58	1.01	72.5	10.89	D.praehensilis:Nigeria:Western D.praehensilis	SRR8451433
ns065_P424	P424	3.46	3.40	3.03	0.04	79.1	6.61	D.praehensilis:Benin:Western D.praehensilis	SRR8451426
ns066_P425	P425	1.63	1.60	1.44	0.02	69.5	3.57	D.praehensilis:Benin:Western D.praehensilis	SRR8451427
ns067_P457	P457	4.21	4.13	3.46	0.12	74.5	8.01	D.praehensilis:Benin:Western D.praehensilis	SRR8451428
ns068_P462	P462	4.33	4.26	3.68	0.08	79.7	7.98	D.praehensilis:Benin:Western D.praehensilis	SRR8451429
ns069_P323	P323	4.22	4.15	3.70	0.05	80.5	7.94	D.praehensilis:Benin:Western D.praehensilis	SRR8451435
ns070_P464	P464	5.29	5.21	4.65	0.05	80.6	9.96	D.praehensilis:Benin:Western D.praehensilis	SRR8451436
ns073_P2990	P2990	2.88	2.84	2.56	0.03	77.6	5.70	D.praehensilis:Benin:Western D.praehensilis	SRR8451409
ns075_P4918	P4918	2.45	2.40	1.82	0.27	72.6	4.33	D.praehensilis:Ghana:Western D.praehensilis	SRR8451415
ns076_P4919	P4919	5.46	5.36	4.04	0.45	79.4	8.79	D.praehensilis:Ghana:Western D.praehensilis	SRR8451414
ns077_P4920	P4920	6.04	5.93	4.63	0.53	80.3	9.95	D.praehensilis:Ghana:Western D.praehensilis	SRR8451413
ns078_P4921	P4921	4.73	4.65	3.73	0.31	79.5	8.11	D.praehensilis:Ghana:Western D.praehensilis	SRR8451412
ns079_P4928	P4928	3.77	3.71	2.99	0.24	78.4	6.57	D.praehensilis:Ghana :Western D.praehensilis	SRR8451407
